# Impacts of land-use and land-cover changes on temperature-related mortality

**DOI:** 10.1097/EE9.0000000000000337

**Published:** 2024-10-21

**Authors:** Anton Orlov, Steven J. De Hertog, Felix Havermann, Suqi Guo, Iris Manola, Quentin Lejeune, Carl-Friedrich Schleussner, Wim Thiery, Julia Pongratz, Florian Humpenöder, Alexander Popp, Kristin Aunan, Ben Armstrong, Dominic Royé, Ivana Cvijanovic, Eric Lavigne, Souzana Achilleos, Michelle Bell, Pierre Masselot, Francesco Sera, Ana Maria Vicedo-Cabrera, Antonio Gasparrini, Malcolm N. Mistry, Multi-Country Multi-City (MCC) Collaborative Research Network

**Affiliations:** a CICERO Center for International Climate Research, Oslo, Norway; b Department of Water and Climate, Vrije Universiteit Brussel, Brussels, Belgium; c Department of Environment, Universiteit Gent, Q-ForestLab, Ghent, Belgium; d Department of Geography, Ludwig-Maximilians-University of Munich, Munich, Germany; e Vrije Universiteit Amsterdam, Institute for Environmental Studies, Amsterdam, Netherlands; f Climate Analytics, Berlin, Germany; g International Institute for Applied Systems Analysis (IIASA), Laxenburg, Austria; h Max Planck Institute for Meteorology, Hamburg, Germany; i Potsdam Institute for Climate Impact Research (PIK), Member of the Leibniz Association, Potsdam, Germany; j Faculty of Organic Agricultural Sciences, University of Kassel, Witzenhausen, Germany; k Department of Public Health Environments and Society, London School of Hygiene & Tropical Medicine, London, United Kingdom; l Climate Research Foundation (FIC), Madrid, Spain; m CIBERESP, Madrid, Spain; n Barcelona Institute for Global Health, Barcelona, Spain; o School of Epidemiology & Public Health, Faculty of Medicine, University of Ottawa, Ottawa, Canada; p Environmental Health Science & Research Bureau, Health Canada, Ottawa, Canada; q Department of Primary Care and Population Health, University of Nicosia Medical School, Nicosia, Cyprus; r School of the Environment, Yale University, New Haven, Connecticut; s School of Health Policy and Management, College of Health Sciences, Korea University, Seoul, Republic of Korea; t Environment & Health Modelling (EHM) Lab, Department of Public Health Environments and Society, London School of Hygiene & Tropical Medicine, London, United Kingdom; u Department of Statistics, Computer Science and Applications “G. Parenti,” University of Florence, Florence, Italy; v Institute of Social and Preventive Medicine, University of Bern, Bern, Switzerland; w Oeschger Center for Climate Change Research, University of Bern, Bern, Switzerland; x Department of Economics, Ca’ Foscari University of Venice, Italy; Universidad de Buenos Aires, Facultad de Ciencias Sociales, Instituto de Investigaciones Gino Germani, Argentina; Department of Epidemiology and Preventive Medicine, School of Public Health and Preventive Medicine, Monash University, Melbourne, Australia, Climate, Air Quality Research Unit, School of Public Health and Preventive Medicine, Monash University, Melbourne, Australia; National Institute of Environmental Health, Chinese Center for Disease Control and Prevention, Beijing, China, School of Public Health and Social Work, Queensland University of Technology, Brisbane, Australia; Department of Pathology, Faculty of Medicine, University of São Paulo, São Paulo, Brazil; INSPER, São Paulo, Brazil; Department of Public Health, Universidad de los Andes, Santiago, Chile; Centro Interdisciplinario de Cambio Global, Pontificia, Universidad Católica de Chile, Santiago, Chile; Department of Environmental Health, School of Public Health, Fudan University, Shanghai, China; Department of Environmental Health, National Institute of Public Health, Mexico; Institute of Atmospheric Physics, Academy of Sciences of the Czech Republic, Prague, Czech Republic, Faculty of Environmental Sciences, Czech University of Life Sciences, Prague, Czech Republic; Institute of Atmospheric Physics, Academy of Sciences of the Czech Republic, Prague, Czech Republic; Department of Family Medicine and Public Health, University of Tartu, Tartu, Estonia; Department of Family Medicine and Public Health, University of Tartu, Tartu, Estonia; Center for Environmental and Respiratory Health Research (CERH), University of Oulu, Oulu, Finland, Medical Research Center Oulu (MRC Oulu), Oulu University Hospital and University of Oulu, Oulu, Finland; Center for Environmental and Respiratory Health Research (CERH), University of Oulu, Oulu, Finland, Medical Research Center Oulu (MRC Oulu), Oulu University Hospital and University of Oulu, Oulu, Finland; Santé Publique France, Department of Environmental and Occupational Health, French National Public Health Agency, Saint Maurice, France; Institute of Epidemiology, Helmholtz Zentrum München – German Research Center for Environmental Health (GmbH), Neuherberg, Germany; IBE-Chair of Epidemiology, LMU Munich, Munich, Germany, Institute of Epidemiology, Helmholtz Zentrum München – German Research Center for Environmental Health (GmbH), Neuherberg, Germany; Department of Hygiene, Epidemiology and Medical Statistics, National and Kapodistrian University of Athens, Athens, Greece, Environmental Research Group, School of Public Health, Imperial College, London, United Kingdom; Department of Hygiene, Epidemiology and Medical Statistics, National and Kapodistrian University of Athens, Athens, Greece; School of Public Health and Community Medicine, University of Gothenburg, Gothenburg, Sweden; University of Münster, Institute of Landscape Ecology, Climatology Research Group, Münster, Germany; Geography and Urban Planning Department, University of Mazandaran, Babolsar, Iran; Technological University Dublin, Dublin, Ireland; UK Health Security Agency, London, United Kingdom; Braun School of Public Health and Community Medicine, The Hebrew University of Jerusalem, Israel; Department of Epidemiology, Lazio Regional Health Service, Rome, Italy; Department of Global Health Policy, Graduate School of Medicine, The University of Tokyo, Tokyo, Japan; Department of Global Environmental Health, Graduate School of Medicine, University of Tokyo, Tokyo, Japan; Department of Environmental Health, Harvard T.H. Chan School of Public Health, Harvard University, Boston, Massachusetts; Department of Environmental Health, National Institute of Public Health, Cuernavaca, Morelos, Mexico; Department of Environmental Health, National Institute of Public Health, Cuernavaca, Morelos, Mexico; National Agency for Public Health of the Ministry of Health, Labour and Social Protection of the Republic of Moldova, Chisinau, Republic of Moldova; National Institute for Public Health and the Environment (RIVM), Centre for Sustainability and Environmental Health, Bilthoven, Netherlands; Norwegian Institute of Public Health, Oslo, Norway; Institute of Tropical Medicine “Alexander von Humboldt,” Universidad Peruana Cayetano Heredia, Lima, Peru; Department of Hygiene, Graduate School of Medicine, Hokkaido University, Sapporo, Japan, School of Tropical Medicine and Global Health, Nagasaki University, Nagasaki, Japan; Department of Global Health Policy, Graduate School of Medicine, The University of Tokyo, Tokyo, Japan; Department of Epidemiology, Instituto Nacional de Saúde Dr. Ricardo Jorge, Lisbon, Portugal; Department of Epidemiology, Instituto Nacional de Saúde Dr. Ricardo Jorge, Lisbon, Portugal, EPIUnit - Instituto de Saúde Pública, Universidade do Porto, Porto, Portugal, Laboratório para a Investigação Integrativa e Translacional em Saúde Populacional (ITR), Porto, Portugal; Faculty of Geography, Babes-Bolay University, Cluj-Napoca, Romania; Department of Environmental Health. Rollins School of Public Health, Emory University, Atlanta, Georgia; Department of Earth Sciences, University of Torino, Italy; Graduate School of Public Health, Seoul National University, Seoul, South Korea; School of Biomedical Convergence Engineering, College of Information and Biomedical Engineering, Pusan National University, Yangsan, South Korea, Institute of Ewha-SCL for Environmental Health (IESEH), Seoul, South Korea; Institute of Environmental Assessment and Water Research (IDAEA), Spanish Council for Scientific Research (CSIC), Barcelona, Spain; Department of Statistics and Computational Research. Universitat de València, València, Spain, CIBERESP, Madrid, Spain; Department of Public Health and Clinical Medicine, Umeå University, Sweden; Swiss Tropical and Public Health Institute, Allschwill, Switzerland, University of Basel, Basel, Switzerland; Environmental and Occupational Medicine, National Taiwan University (NTU) College of Medicine and NTU Hospital, Taipei, Taiwan, National Institute of Environmental Health Science, National Health Research Institutes, Zhunan, Taiwan, Graduate Institute of Environmental and Occupational Health Sciences, NTU College of Public Health, Taipei, Taiwan; National Institute of Environmental Health Science, National Health Research Institutes, Zhunan, Taiwan; Department of Epidemiology and Preventive Medicine, School of Public Health and Preventive Medicine, Monash University, Melbourne, Australia, Climate, Air Quality Research Unit, School of Public Health and Preventive Medicine, Monash University, Melbourne, Australia; Department of Quantitative Methods, School of Medicine, University of the Republic, Montevideo, Uruguay; Department of Environmental Health, Harvard T.H. Chan School of Public Health, Harvard University, Boston, Massachusetts; Institute of Research and Development, Duy Tan, University, Da Nang, Vietnam, Department of Environmental Health, Faculty of Public Health, Department of Environmental Health, University of Medicine and Pharmacy at Ho Chi Minh City, Ho Chi Minh City, Vietnam; Department of Environmental Health, Faculty of Public Health, Department of Environmental Health, University of Medicine and Pharmacy at Ho Chi Minh City, Ho Chi Minh City, Vietnam; Queen Mary University of London, Malta Campus, Triq l-Arċisqof Pietru Pace Victoria, Malta

**Keywords:** Land-use and land-cover change, Sustainable land use, Deforestation, Temperature, Mortality

## Abstract

**Background:**

Land-use and land-cover change (LULCC) can substantially affect climate through biogeochemical and biogeophysical effects. Here, we examine the future temperature–mortality impact for two contrasting LULCC scenarios in a background climate of low greenhouse gas concentrations. The first LULCC scenario implies a globally sustainable land use and socioeconomic development (sustainability). In the second LULCC scenario, sustainability is implemented only in the Organisation for Economic Cooperation and Development countries (inequality).

**Methods:**

Using the Multi-Country Multi-City (MCC) dataset on mortality from 823 locations in 52 countries and territories, we estimated the temperature–mortality exposure–response functions (ERFs). The LULCC and noLULCC scenarios were implemented in three fully coupled Earth system models (ESMs): Community Earth System Model, Max Planck Institute Earth System Model, and European Consortium Earth System Model. Next, using temperature from the ESMs’ simulations and the estimated location-specific ERFs, we assessed the temperature-related impact on mortality for the LULCC and noLULCC scenarios around the mid and end century.

**Results:**

Under sustainability, the multimodel mean changes in excess mortality range from −1.1 to +0.6 percentage points by 2050–2059 across all locations and from −1.4 to +0.5 percentage points by 2090–2099. Under inequality, these vary from −0.7 to +0.9 percentage points by 2050–2059 and from −1.3 to +2 percentage points by 2090–2099.

**Conclusions:**

While an unequal socioeconomic development and unsustainable land use could increase the burden of heat-related mortality in most regions, globally sustainable land use has the potential to reduce it in some locations. However, the total (cold and heat) impact on mortality is very location specific and strongly depends on the underlying climate change scenario due to nonlinearity in the temperature–mortality relationship.

**What this study adds:**

Numerous environmental epidemiological studies have investigated the temperature-related mortality impact of changes in global greenhouse gas concentrations. However, more scientific evidence is needed on the temperature-related impacts of land-use and land-cover change (LULCC) on human health. An effective climate policy to achieve the targets of the 2015 Paris Agreement requires a substantial transformation in the land sector. Based on recently developed land-use scenarios, this interdisciplinary study contributes to the literature by assessing the temperature-related mortality impacts induced by LULCC using three Earth system models and the most comprehensive exposure–response functions.

## Introduction

Climate change can substantially affect human morbidity and mortality. Empirical evidence suggests that climate change alone would decrease cold-related deaths and increase heat-related deaths.^1^ The total impact on temperature-related mortality is often unclear as it depends on the magnitude of warming and the temperature–mortality relationship, which is generally found to be U-shaped (convex) and region specific. Future global-scale projections of temperature-related mortality, which are typically based on regression models estimated using observed location-specific temperature–mortality data and projected using temperature simulated by global climate models, provide spatially heterogeneous results regarding the total climate-induced impact on mortality across regions.^1,2^

Most global-scale epidemiological studies focusing on human health impacts of climate change, such as Gasparrini et al^1^ and Vicedo-Cabrera et al,^3^ investigate the future temperature-related impact on mortality for different global greenhouse gas (GHG) emissions trajectories using the Representative Concentration Pathway (RCP) framework.^4^ However, less is known about the temperature-related impacts on human health induced by land-use and land-cover change (LULCC), especially on a global scale.^5^ Few impact studies have assessed the deforestation-induced impact on heat stress and labor capacity,^6–9^ and even fewer studies have specifically investigated the regional impact of land-use change on mortality. For example, Wolff et al^10^ assessed the impact of deforestation and climate change on mortality and unsafe work conditions in Berau (Indonesia) and found that deforestation in Berau increased heat-related deaths by 7.3%–8.5% (or 101–118 additional deaths per year) in 2018.

At the same time, agriculture, forestry, and other land use (AFOLU) is considered an important sector in mitigation policy. An effective climate policy to achieve the Paris Agreement targets would require a substantial transformation in the AFOLU sector (e.g., reduced deforestation and increased reforestation). Substantial changes are anticipated to meet the needs of food and fiber for an increasing and more affluent population while requiring climate services such as CO_2_ removal. The climate response to land-use changes is complex, acting via various mechanisms and at local to global scales. Apart from the biogeochemical effect (i.e., release or uptake of CO_2_ and other greenhouse gases to or from the atmosphere), LULCC can also induce biogeophysical effects, such as alteration of local energy and water fluxes at the land surface, and their interaction with large-scale atmospheric dynamics.^12^ Climate model simulations and satellite-based observations show that the biogeophysical effects of LULCC could substantially affect the climate.^13,14^ Moreover, the biogeophysical effects of LULCC can not only change the climate locally but can also affect the climate in regions where no LULCC occurred through atmospheric circulation processes.^14–16^

Thus, LULCC can have a substantial impact on climate, which in turn can translate into a modification of the temperature-related mortality burden. Using the output from three fully coupled Earth system models (ESMs) and exposure–response functions (ERFs) derived using the most globally comprehensive mortality dataset to date, we assessed how LULCC could affect the human mortality in different world regions through LULCC-induced changes in temperature (i.e., biogeochemical and biogeophysical effects). Potential changes in vulnerability and adaptation are out of scope of this study.

## Data and methods

### Earth system models simulations

**Figure 1.**
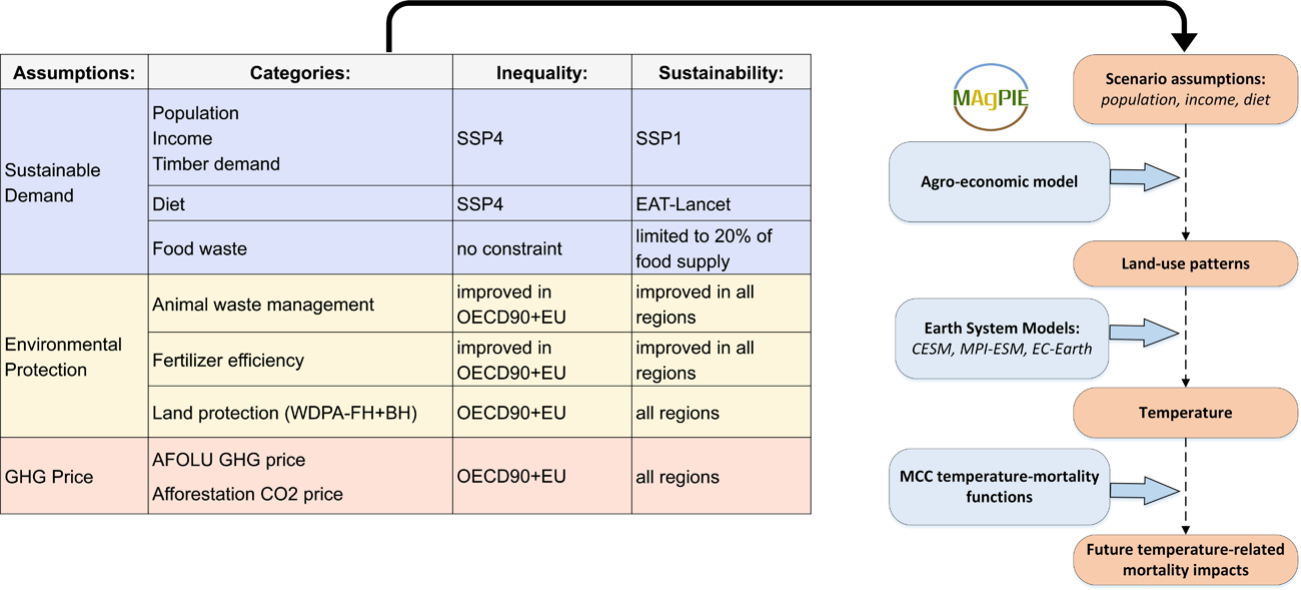
Schematic presentation of the two LULCC scenarios (inequality and sustainability) and modeling interface. The scenarios are described in detail in Humpenöder et al.^22^

Our analysis is based on three simulations conducted by each of the three ESMs: the Community Earth System Model (CESM) Version 2.1.3,^17^ the Max Planck Institute Earth System Model (MPI-ESM) Version 1.2,^18^ and the European Consortium Earth System Model (EC-Earth) Version 3.^19^ For each ESM, a control simulation is run for the time period of 1980–2099 using GHG concentrations from the RCP1.9 scenario as forcing data.^20^ RCP1.9 describes a climate trajectory where an increase in the radiative forcing from GHG is limited to 1.9 W/m^2^ above preindustrial levels, which is associated with a global mean temperature increase of below 1.5 °C. For this control simulation, land use and land cover are kept constant at 2014 levels of the Land-Use Harmonization (LUH2) dataset (hereafter, the control simulation is referred to as noLULCC scenario).^21^ Building up on the noLULCC scenario, ESMs implement two contrasting land-use and land-cover scenarios, referred to as sustainability and inequality (Figure 1), which are derived from the agroeconomic model MAgPIE described in Humpenöder et al^22^ and are run for the time period of 2015–2099.

The sustainability scenario assumes a global implementation of (1) pricing of GHG emissions from the AFOLU sector; (2) strict environmental protection measures (for land, water, and nitrogen input), and (3) an inclusive economic development (i.e., low population growth, and high economic growth) together with shifts to healthy diets. In the sustainability scenario, socioeconomic assumptions including population and income follow the Shared Socioeconomic Pathway 1 (SSP1),^23,24^ while dietary patterns transition to the EAT-Lancet planetary health diet by 2050.^25^

In contrast, the inequality scenario describes a world where sustainable land use is implemented only in the Organisation for Economic Cooperation and Development (OECD) countries. In this scenario setting, (1) AFOLU GHG emission pricing and (2) strict environmental protection remain limited to OECD countries. Furthermore, socioeconomic assumptions including dietary patterns in the inequality scenario follow SSP4,^26^ which is characterized by highly unequal developments between high-income countries (i.e., low population growth, high economic growth) and middle- and low-income countries (i.e., high population growth, low economic growth). All simulations are initialized thrice, thereby building three ensemble members for each ESM. This is done by initiating the ESM simulations from three different historical simulations (histctl) in MPI-ESM and EC-EARTH, in CESM only two such simulations were available thus a third member was generated by adding a slight perturbation to the initial conditions.

The land-use patterns simulated by the MAgPIE model (Humpenöder et al^22^) were harmonized within the common data format used in the Land Use Harmonisation (LUH) project (Hurtt et al^21^) and then implemented in the ESMs to simulate the associated climatic responses (Figure S1; http://links.lww.com/EE/A302). Specifically, the ESMs’ simulated 3-hourly near-surface temperatures were aggregated to daily mean levels before assessing the impacts on future mortality (described further in “Excess mortality due to cold, heat, and total (cold plus heat)” section). It is important to emphasize the choice of the RCP1.9 scenario that forms the basis of the ESMs’ simulations in our analyses. The radiative forcing levels (or greenhouse gas concentrations) defining this pathway limit global warming to levels compatible with the Paris Agreement and are lower than other RCP scenarios frequently used in climate science, such as RCPs 2.6, 4.5, 3.7, and 8.5. This means that the temperature response to land-cover changes relative to global warming in the sustainability and inequality scenarios is larger than in higher-emission scenarios.

### Description of the epidemiological framework

#### Mortality data

**Figure 2.**
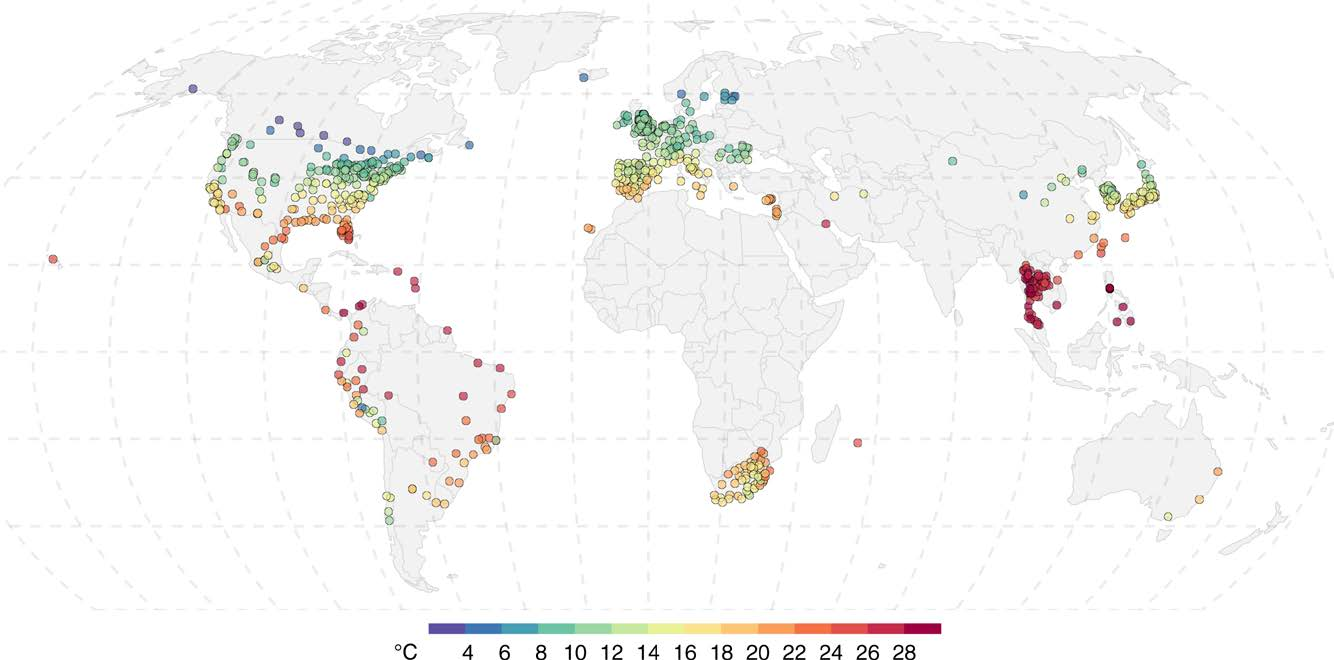
Average daily mean temperature (°C) at the 823 MCC locations across 52 countries and territories in five inhabited continents used in this study. The daily mean temperature is computed using surface observations from the MCC data and averaged over the location-specific time periods shown in Table S1; http://links.lww.com/EE/A302.

The historical temperature–mortality relationship is estimated using a time-series regression model fitted on the daily mortality data collected by the Multi-Country Multi-City (MCC) Collaborative Research Network (https://mccstudy.lshtm.ac.uk/). The MCC data used in this study include: (1) a total of 121,422,567 daily mortality counts from all causes or nonexternal causes (International Classification of Diseases, ICD-9: 0-799; ICD-10: A00-R99) across 52 countries and territories, gathered over 823 locations (mainly cities and in some instances wider geographic scales such as districts, smaller regions and prefectures) (Figure 2 and Table S1; http://links.lww.com/EE/A302), and (2) daily near-surface mean air temperature (*T*_obs,_ units: °C) gathered from the local weather stations. Since the MCC data are available for different time intervals across countries, the daily mortality time series in this study span largely overlapping time periods between 1985 and 2021, with the shortest being 4 years (2013–2016) for Panama and the longest being 36 years (1985–2020) both for Israel and Japan. In addition, other location-specific meta-variables gathered by the MCC and used in this study include weather indices derived from the series of *T*_obs_ (e.g., average and range of temperature across the whole study period), climatological zones based on the Köppen–Geiger classification,^27^ and country-specific gross domestic product (GDP) per capita. Further details on the MCC data collection and summary statistics are provided in Table S1 and S2; http://links.lww.com/EE/A302, as well as documented in previous studies.^1,28^

#### Two-stage empirical framework

We applied the well-established two-stage modeling framework to estimate the location-specific temperature–mortality associations across the 823 locations covering a wide range of climates and socioeconomic conditions. The methodology is described in detail in previous studies^1,2,11,28,29^ and elaborated further in supplementary information. In brief, location-specific temperature–mortality association was first estimated through time-series analyses, with quasi-Poisson regression, distributed lag nonlinear models for temperatures, and additional terms to capture confounding. Second, the location-specific associations were then pooled with a multivariate random meta-regression model that includes the meta-predictors described earlier. This meta-regression model is then used to derive the best linear unbiased predictions (BLUPs) for each location.^3^ The flexible modeling framework allows for nonlinear/lagged responses, separation of effects due to cold/heat and moderate/extreme temperature, and heterogeneity of estimates at various geographical levels. Further details on the empirical framework are provided in Supplementary Information; http://links.lww.com/EE/A302.

#### Excess mortality due to cold, heat, and total (cold plus heat)

Next, utilizing the derived location-specific exposure–response relationships, we computed the cold, heat, and total (cold plus heat) excess mortality (EM) following a method described in previous works.^28–30^ EM is defined as the number of deaths and the related fraction as the proportion of deaths attributed to cold, heat, and total. For each location-day combination, we computed the number of cold- and heat-related deaths based on the ESM scenario-specific temperature series, daily baseline mortality for the total population that is assumed to be constant over time, and the estimated temperature–mortality association represented by the location-specific BLUPs.^30^ We then estimated the total number of cold- and heat-related deaths in each location across the study period by summing the daily mortality contributions when the temperature on a specific day was lower (higher) than the location-specific minimum mortality temperature (MMT). The fraction of excess deaths due to cold, heat, or total is computed using the total number of all-cause deaths in the historical time period as the denominator (more details in Supplementary Information; http://links.lww.com/EE/A302). We quantified the uncertainty of the estimates by generating 1000 samples of the coefficients of the BLUPs (representing the association) through Monte Carlo simulations, assuming a multivariate normal distribution for the estimated spline model coefficients. We obtained empirical confidence intervals corresponding to the 2.5th and 97.5th percentiles of the empirical distribution of the cold- and heat-related mortality impacts across coefficients (See Table S2; http://links.lww.com/EE/A302 for further details of the EM and associated fractions at the city, country, and wider-regional levels).

#### Projections of future excess mortality under no adaptation or population changes

**Figure 3.**
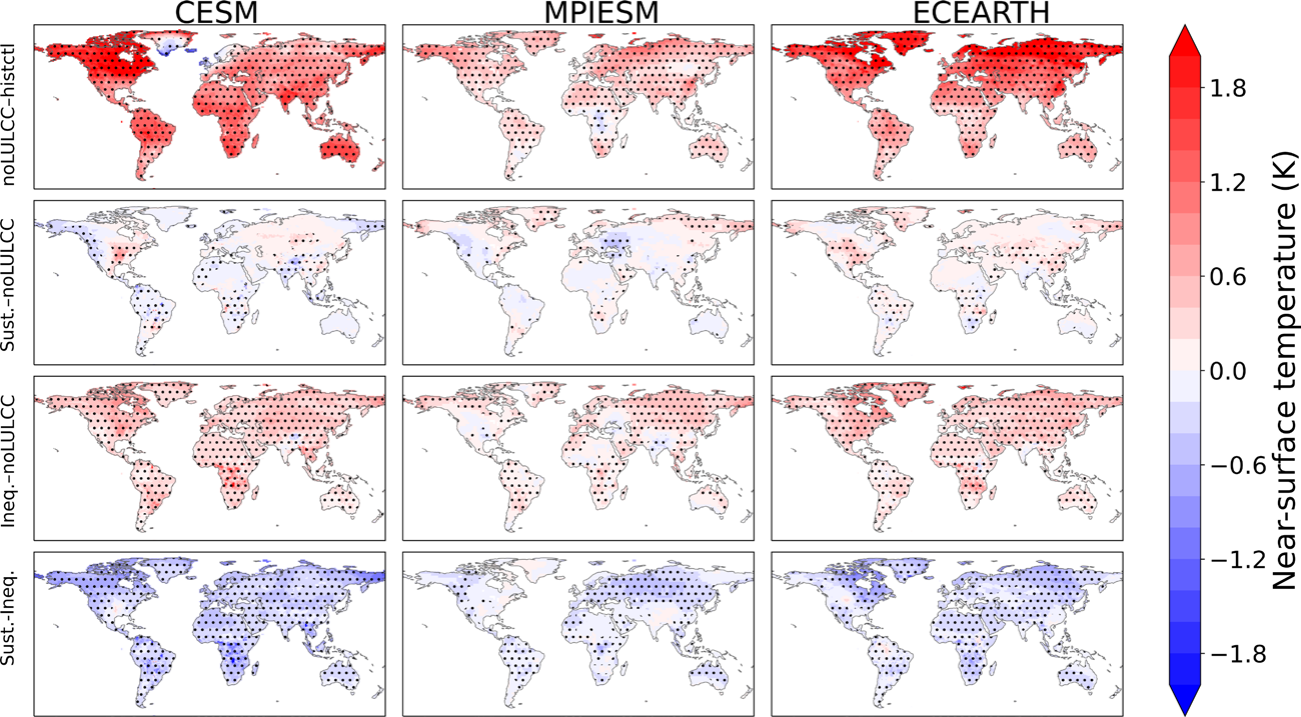
Change in near-surface mean air temperature (°K) by 2069–2099. The noLULCC scenario is compared to the historical control (1980–2014). The sustainability and inequality scenarios are compared to the noLULCC scenario (2069–2099). The dots indicate the consistency in terms of the sign of temperature response across the three individual ensemble members of the ESMs.

Following the approach adopted by Gasparrini et al^1^ and Vicedo-Cabrera et al^3^ and explained in-depth by Vicedo-Cabrera et al,^11^ we then combined the estimated temperature–mortality association with the temperature series from the three ESMs and three scenarios (described in “Earth system models simulations” section). Specifically, we computed the cumulative cold- and heat-related, and total EM from 1980 until 2099 by extracting the ESM scenario combination of the daily temperature series for each of the studied locations in the period 1980–2099, and then calibrating them to the observed historical temperature time series using the trend preserving bias-adjustment approach of Hempel et al.^31^ We then projected the daily mortality series by computing the average observed counts for each day of the year repeated along the same projection period (1980–2099) (Supplementary Information; http://links.lww.com/EE/A302 for further details on the calibration step and construction of the baseline mortality time series). The projected deaths where the corresponding days have a temperature below (higher) than the MMT are deemed as cold-related (heat-related) EM, with the results reported in Results section expressed as ESM and scenario-specific fraction of EM. For instance, in the case of the noLULCC scenario, we calculated the changes in EM under noLULCC relative to the historical control simulation 10-year average in 1980–1989 for each ESM. Similarly for the inequality and sustainability scenarios, the changes in EM are calculated relative to the noLULCC scenario (hereafter: inequality–noLULCC and sustainability–noLULCC, respectively). Our estimates assume both constant demographic structure and population size. The temperature–mortality analyses in this study were done with R software (version 4.3.2; R Foundation for Statistical Computing, Vienna, Austria)^32^ using packages dlnm (ver 2.4.7)^33^ and mixmeta (1.2.0).^34^ All graphics in the study, including those in the Supplementary Information; http://links.lww.com/EE/A302 (except Figures 1 and 3, which were created in Microsoft Visio and Python package matplotlib),^35^ have been generated using R package ggplot2 (ver 3.4.4).^36^

## Results

We discuss the results for mid and end century mortality impacts derived using both individual ESMs and the multimodel mean (ensemble of three ESMs). A comprehensive set of decadal aggregated results spanning 2020–2099 for each combination of ESM and scenario are further provided in Table S2; http://links.lww.com/EE/A302.

### No land-use and land-cover changes

Under noLULCC, the three ESMs simulate an increase in global mean temperature relative to the current climatic conditions (1985–2015) because of an increase in GHG concentrations from fossil fuel consumption (Figure 3). CESM shows an increase in global mean temperature of 1.05 °C by the end of the century relative to 1985–2015, for EC-Earth, it is 1.02 °C, and for MPI-ESM, it is 0.35 °C (Table S3; http://links.lww.com/EE/A302). For EC-Earth and MPI-ESM, almost all locations experience an increase in temperature, whereas for CESM, some European cities, especially in Northern and Western Europe, show a regional cooling effect (Figures S2 and S3; http://links.lww.com/EE/A302). For CESM, cooling over Northern and Western Europe results from changes in the Atlantic Meridional Overturning Circulation. Overall, CESM and EC-Earth show larger increases in temperature compared to MPI-ESM because these two ESMs have a higher equilibrium climate sensitivity.

**Figure 4.**
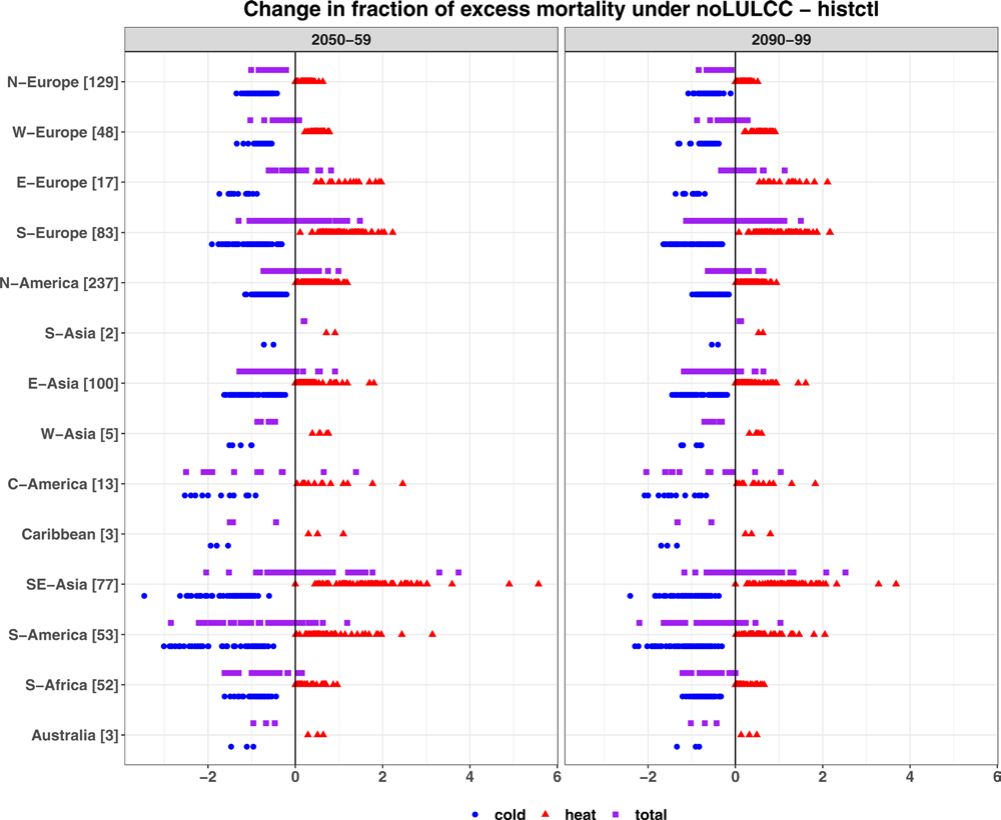
Multimodel mean changes in the fraction of total (cold and heat) excess mortality (percentage points) in 2050–2059 (left panel) and 2090–2099 (right panel) under noLULCC relative to a historical period of 1980–1989. The blue circles, red triangles, and purple rectangles, respectively, represent cold-related, heat-related, and total EM-derived estimates for 823 MCC locations across 14 geographic regions. The world regions are ordered by latitude on the *Y*-axis. The numbers following the region names on *Y*-axis show the number of MCC locations. Uncertainty in the empirical distribution of EM (i.e., 2.5th and 97.5th percentiles) are estimated using Monte Carlo simulations and shown in Table S2; http://links.lww.com/EE/A302.

Our results for noLULCC (RCP1.9) suggest that global warming would lead to a decrease in the cold-related EM and an increase in the heat-related EM across the locations examined here. Results show that the multimodel mean changes in total EM relative to 1980–1989 range from −2.8 to +3.7 percentage points across all locations by 2050–2059, and from −2.2 to +2.5 percentage points by 2090–2099 (Figure 4). For some regions, the responses of total EM are qualitatively consistent in terms of sign across locations (Figure 4 and Figures S8 and S9; http://links.lww.com/EE/A302). For example, the multimodel mean shows a reduction in total EM by 2090–2099 in most locations of Australia, South Africa, Caribbean, West and East Asia, Northern and Western Europe (Figure 4). Under noLULCC, a reduction in total EM is driven by a larger reduction in the cold-related EM (i.e., an increase in the heat-related EM being outnumbered by a decrease in the cold-related EM). This is likely due to a shift in the future temperature distribution resulting in a larger decline in exposure to days with daily mean temperature below the location-specific MMTs, compared to a corresponding marginal increase in days with daily mean temperature above their respective MMTs. Figure S19; http://links.lww.com/EE/A302 illustrates this using Santiago (Chile) as an example. For other regions, such as Southern and Eastern Europe, North America, and South-East Asia, the three ESMs show mixed temperature-induced impacts on total EM across locations within a region (Figures S8 and S9; http://links.lww.com/EE/A302).

Comparing across the three ESMs, EC-Earth shows a more consistent reduction in total EM in locations of Northern and Western Europe compared to CESM. For the MPI-ESM simulations, which show smaller temperature increases, the temperature–mortality impacts are quantitatively less pronounced and less consistent in terms of sign compared to CESM and EC-Earth. For instance, over almost all locations, the change in the fraction of total EM does not exceed 1 percentage point relative to 1980–1989. Similar to EC-Earth, MPI-ESM shows a reduction in total EM across almost all locations of Northern Europe, whereas Eastern Europe experiences a relatively consistent increase in total EM.

### Sustainability

Under the sustainability scenario, which implies global sustainable land use, AFOLU GHG emission pricing, and inclusive socioeconomic development, the temperature responses are generally less pronounced and less consistent in terms of signs compared to the noLULCC scenario (Figure 3). CESM shows a reduction in global mean temperature of −0.07 °C by the end century, while for EC-Earth and MPI-ESM, the global mean temperature increases by +0.07 °C and +0.05 °C, respectively (Table S3; http://links.lww.com/EE/A302). Moreover, some regions show inconsistency in the temperature response across the three ensemble members of each ESM (represented by the dots in Figure 3.). This likely indicates that the temperature response can be driven mainly by internal climate variability rather than the climate signal resulting from the simulated LULCC. For a few regions, the temperature responses are relatively consistent in terms of signs across locations (Figures S4 and S5; http://links.lww.com/EE/A302). For example, all three ESMs show a reduction in temperature in most locations of Europe, except Western Europe where CESM shows mixed mortality impacts. Furthermore, both CESM and EC-Earth show a relatively consistent increase in temperature in most locations of South and East Asia. In contrast to the other two ESMs, CESM shows a more consistent reduction in temperature in almost all locations of South America and South-East Asia.

**Figure 5.**
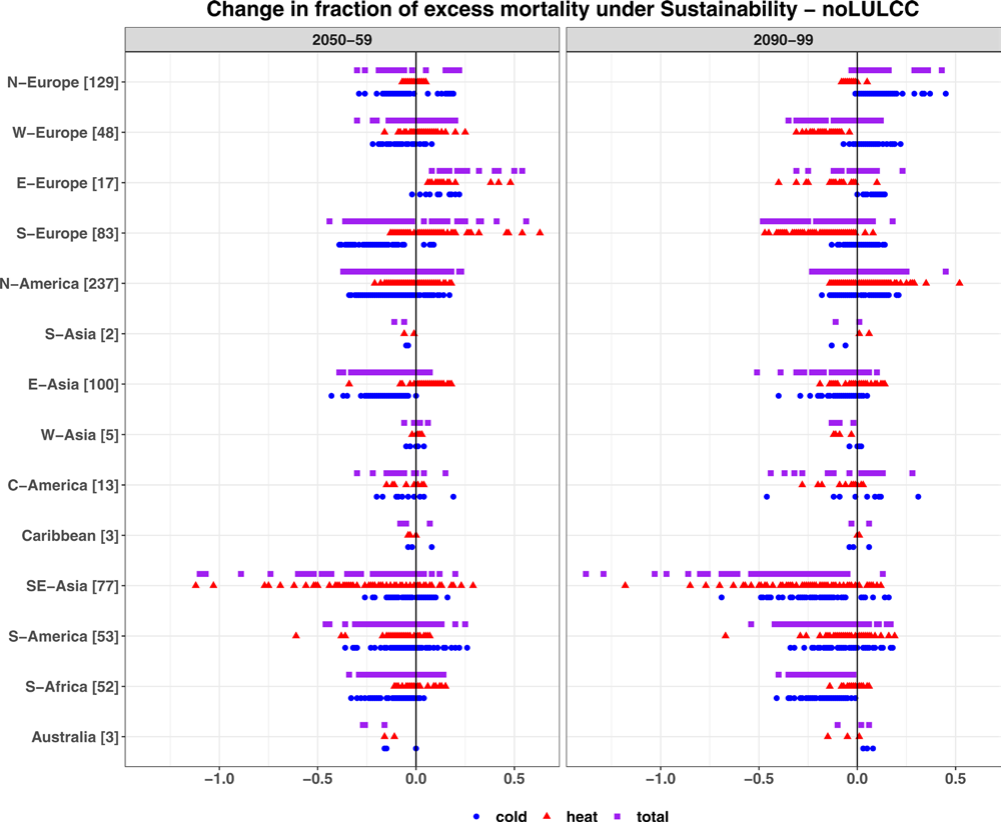
Same as Figure 4 but for the sustainability scenario relative to the noLULCC scenario by 2050–2059 (left panel) and 2090–2099 (right panel). The *X*-axis ranges in this figure differ from those in Figure 4.

Because the temperature responses as well as the temperature–mortality associations differ by location, the temperature–mortality impacts of globally sustainable LULCC are also mixed and location specific in terms of sign and magnitude (Figure 5 and Figures S10 and S11; http://links.lww.com/EE/A302). Overall, under the sustainability scenario, results show that the multimodel mean changes in total EM relative to the noLULCC scenario by 2050–2059 range from −1.1 to +0.6 percentage points across all locations and from −1.4 to +0.5 percentage points by 2090–2099. By 2050–2059, Eastern Europe is the only region for which the multimodel mean shows an increase in total EM in all locations, driven by an increase in cold- and heat-related mortality. However, by 2090–2099, the impact on total EM is less consistent across locations since the heat-related EM decreases in most locations. For South Asia and Australia, the multimodel mean shows a reduction in total EM by 2050–2059 in all locations, whereas the impacts on total EM are mixed by 2090–2099. By 2090–2099, South Africa, South-East and West Asia, and Southern Europe show a reduction in total EM in most locations, whereas Northern Europe experiences an increase in total EM. Compared to CESM and EC-Earth, MPI-ESM generally shows a tendency toward a reduction in total EM in most locations.

### Inequality

The inequality scenario implies unequal socioeconomic development between the Global North and the Global South. In addition, sustainable land use and AFOLU GHG emission pricing remain limited to OECD countries. Under the inequality scenario, the three ESMs used in this study simulate an additional global warming effect relative to the noLULCC scenario, which is mainly driven by deforestation (Figure 3). CESM and EC-Earth show an additional increase in global mean temperature of 0.29 °C by the end century, and for MPI-ESM, it is 0.17 °C (Table S3; http://links.lww.com/EE/A302). An increase in temperature in most locations can be noted for all three ESMs (Figures S6 and S7; http://links.lww.com/EE/A302). A notable contrast though can be seen for MPI-ESM, where many locations in North America experience a decline in temperature. This is most likely induced by the evaporative cooling with a relatively small role for albedo-induced warming in MPI-ESM compared to CESM and EC-Earth. Also, CESM shows a reduction in temperature for many locations of Europe, especially in Southern Europe, and South-East Asia.

**Figure 6.**
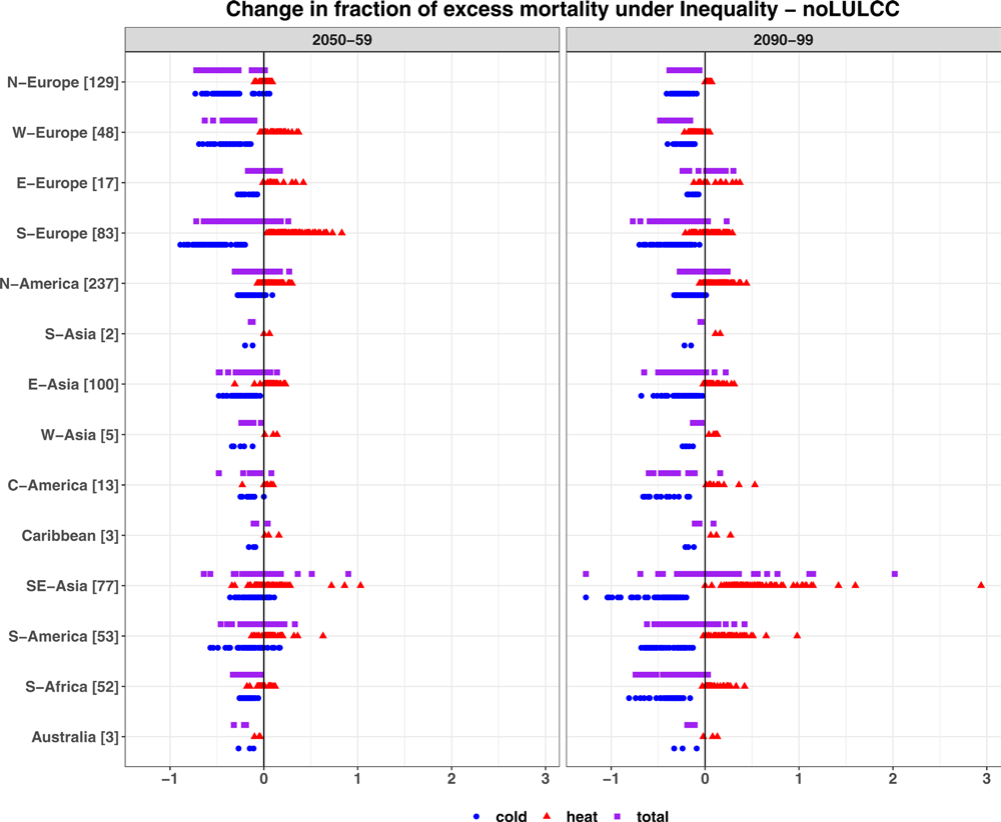
Same as Figure 4 but for the inequality scenario relative to the noLULCC scenario by 2050–2059 (left panel) and 2090–2099 (right panel). The *X*-axis ranges in this figure differ from those in Figures 4 and 5.

As a result of increased global warming under inequality, for most locations, there are more heat-related and less cold-related EM compared to the noLULCC scenario (Figure 6 and Figures S12 and S13; http://links.lww.com/EE/A302), findings that are in line with earlier global-scale studies for RCP2.6.^1^ Overall, results indicate that the multimodel changes in total EM across all locations range from −0.7 to +0.9 percentage points by 2050–2059 and from −1.3 to +2 percentage points by 2090–2099. For some regions, three ESMs show a relatively consistent impact on total EM. For example, by both mid and end century, the multimodel mean shows a reduction in total EM in Northern and Western Europe, East and West Asia, Central America, Australia, and South Africa across most locations within those regions. These results here can again be explained by the low warming scenario (RCP1.9), illustrated in Figure S20; http://links.lww.com/EE/A302 using Asuncion (Paraguay) as an example. For other regions, such as Eastern Europe and South-East Asia, the mortality impacts are mixed in terms of signs across locations. Compared to two other ESMs, MPI-ESM shows a more consistent reduction in total EM in most regions, except South-East Asia and Eastern Europe.

### Influence of land-use and land-cover change on future mortality impacts

Having discussed the location-specific temperature–mortality impacts above, we now compare the regionally aggregated total EM across the three scenarios and ESMs. The comparison enables us to draw insights into the relevance of LULCC-induced impacts on temperature-related mortality when compared to the impacts of changes in fossil fuel GHG concentrations consistent with RCP1.9. Although many world regions are either underrepresented or not represented in the MCC dataset (e.g., India and Africa), thus making it difficult to draw any robust conclusion on the global mortality response, the regionally aggregated mortality impacts nevertheless facilitate a consistent comparison across the three scenarios and ESMs.

**Figure 7.**
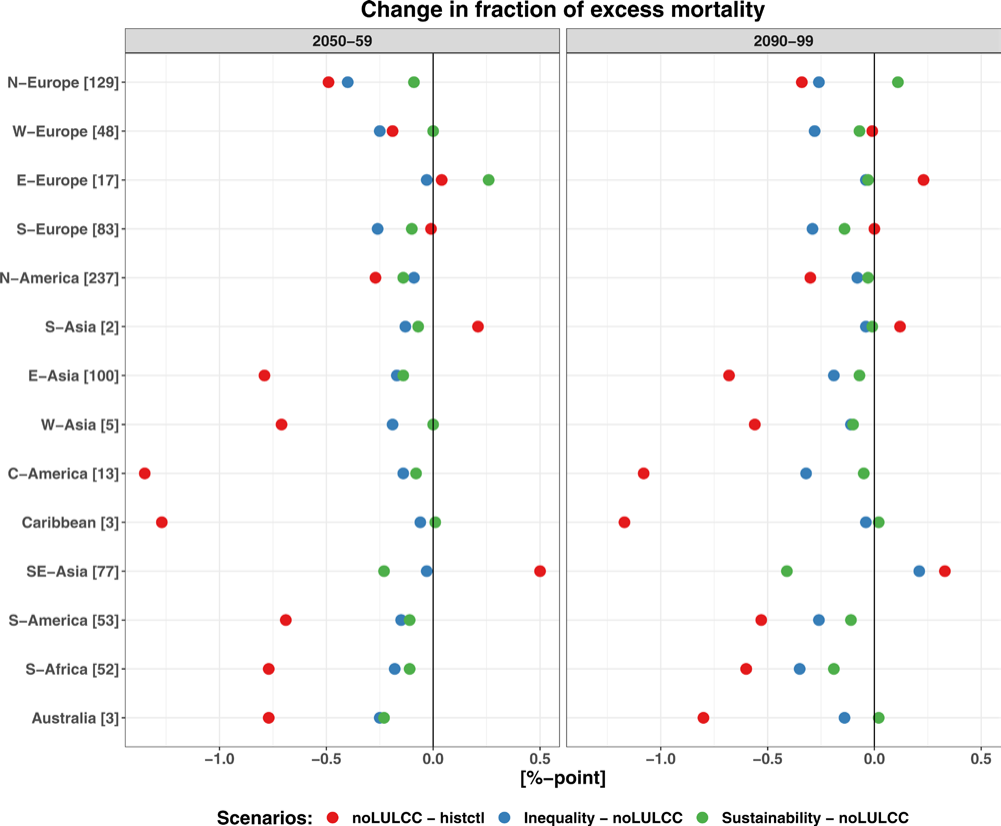
Regionally aggregated multimodel mean change in the fraction of total excess temperature-related mortality fractions (percentage points) in 2050–2059 (left panel) and 2090–2099 (right panel). The red, blue, and green circles show the mortality impacts for the noLULCC, inequality, and sustainability scenarios, respectively, grouped by 14 geographic regions. The numbers following the region names on the *Y*-axis show the number of MCC locations. The regions with a small number of locations (e.g., S-Asia and Caribbean) are underrepresented.

Under noLULCC, CESM and EC-Earth show a reduction in total EM in many low-latitude regions (except South-East Asia) by 2090–2099 relative to 1980–1989, because of a decrease in cold-related deaths outnumbering an increase in heat-related deaths as highlighted earlier in “noLULCC” section (Figure 7 and Figures S14–S17; http://links.lww.com/EE/A302). Results show that the multimodel mean changes in total EM across all locations vary from −1.2 to +0.3 percentage points by 2090–2099. For high-latitude regions, the mortality impacts are less consistent across ESMs. Furthermore, for both CESM and EC-Earth, global warming from fossil fuels consumption (noLULCC) shows a substantially larger impact on total EM, especially in low latitudes, than the LULCC scenarios. An exception is South-East Asia in CESM simulations where the inequality scenario shows a larger mortality impact compared to the noLULCC scenario.

In contrast, for MPI-ESM, the regionally aggregated temperature–mortality impacts under the noLULCC scenario are smaller compared to CESM and EC-Earth. Also, for MPI-ESM, which has the lowest equilibrium climate sensitivity among the three ESMs, the LULCC-induced impact on temperature-related mortality tends to be as large as or, for some regions, even larger than under the noLULCC scenario. For example, under the inequality scenario implemented in MPI-ESM, South America experiences a much larger reduction in total EM compared to the noLULCC scenario.

## Discussion

The primary motivation of our study was to examine how temperature-related human mortality can evolve under future LULCC scenarios, a daunting research question to the best of our knowledge not explored to date at a global scale. Here, we examined the future temperature-related impacts on mortality under two contrasting LULCC scenarios and under a low emission scenario (RCP1.9), using three ESMs and a well-established empirical modeling framework employed in environmental epidemiology. Our estimates of future mortality responses do not account for changes in demographic and population patterns nor vulnerability and adaptation, thus allowing us to isolate and assess how LULCC-induced climate effects can modulate future EM from the combined effects of cold and heat. Note that the temperature response resulting from the LULCC is driven by changes in demographics and population through SSP-driven changes in food demand (see “Earth system models simulations” section).

Results of the ESM simulations show substantial quantitative differences in the temperature response across three ESMs, which could indicate the uncertainties in climate modeling of biogeochemical and biogeophysical effects of LULCC.^8,14^ Further understanding of LULCC-induced impacts on climate as well as model development is needed to address these uncertainties. Nonetheless, results of the ensemble mean of the three ESMs show a change in the regionally aggregated total EM by the middle and end of the century that are broadly in line with an earlier global-scale study employing a similar low emission (RCP2.6) scenario.^1^ Our results indicate that global warming induced by burning of fossil fuels consistent with RCP1.9 (noLULCC) as well as globally unsustainable land use (inequality) can lead to a reduction in the total EM by the end of the century relative to 1980–1989, across most regions examined in this study. While counter-intuitive at first sight, especially for locations in low latitudes, this reduction in the total EM is primarily due to the decline in the number of cold-related deaths in the future outnumbering the corresponding increase in the number of heat-related deaths, as discussed in “noLULCC” section and illustrated in Figures S18, S19 and S20; http://links.lww.com/EE/A302. This, in turn, is largely due to a decline in the exposure to days below present-day MMT temperatures. Moreover, due to acclimatization in tropical regions, the values of MMT are typically higher in low latitudes than in high-latitude regions. The limited net negative impacts of temperature-related mortality and in some cases a marginal net positive benefit would therefore be expected under RCP1.9, an emission pathway that corresponds to the goal of the Paris Agreement to limit global warming to below 1.5 °C. As previous studies have demonstrated an increase in total EM globally under higher-emission scenarios (e.g., RCPs 4.5 and 8.5),^1^ the reductions in total EM found in this study for noLULCC and inequality scenarios are most likely to reverse, showing an increased burden of total EM. Investigating the LULCC-induced impacts on temperature-related mortality under more higher warming scenarios using a larger ensemble of ESMs is recommended for future research.

Notable limitations in the methodology applied in our study are worthy of mention. We focus here on the main caveats and refer the readers to some other persistent challenges and limitations discussed in Orlov et al.^5^ First, due to the lack of reliable daily mortality records in many parts of the world, a number of inhabited regions either remain underrepresented (e.g., Africa) or are completely missing (e.g., India) in our epidemiological assessment. While efforts are ongoing within the MCC network to gather mortality data across the wider locations in such countries, the limited regional coverage in our analysis does not allow us to draw robust conclusions on the global mortality response. Second, the ERFs for temperature-related mortality were estimated at the city level across the majority of locations, meaning that the temperature–mortality response of the rural population is largely excluded in our analysis. LULCC-induced impacts on temperature might have a stronger impact on rural populations than in the urban context. Ongoing efforts for more representative (mortality) data collection from smaller towns or rural regions should help to address these limitations in future research. Third, the meteorological exposure variable used in our study was air temperature, though other climatic variables, such as humidity, radiation, and wind speed, might also have a substantial impact on health and could be affected by LULCC, especially humidity.^37^ Exploring the mortality response to changes in those climatic variables in future LULCC scenarios is recommended for further research. The applied epidemiological model is estimated using the data on all-cause mortality, while the impacts on cause-specific mortality, data that are generally even harder to obtain, might significantly differ. In addition to temperature-related impacts, LULCC could also indirectly affect human health through changes in zoonoses and biomass burning, which would amplify adverse health impacts of unsustainable land use.^38,39^ Finally, our estimates of changes in EM do not account for (1) potential location-scale adaptation or acclimatization to both cold and heat in the future, (2) changes in future population and demographics (e.g., population aging), (3) changes in vulnerability derived from changes in LULCC, and (4) changes in exposure–response relationship due to changes in future urban green space; all active areas of research currently pursued by the climate–health impact modelers.

Nonetheless, our interdisciplinary study employing a state-of-the-art epidemiological framework brings in several strengths with a potential of making a notable contribution to the climate–health literature. To the best of our knowledge, this is the first global-scale study to specifically account for LULCC-induced climate effects in projecting future temperature-related mortality, by utilizing multiple fully coupled ESMs and LULCC scenarios. Our study benefits from the most comprehensive mortality dataset currently gathered: spanning 823 locations across 52 countries and territories in five inhabited continents, thus enabling us to assess not only the local-scale climate signal from the different LULCC scenarios but also the vulnerability of each studied population through localized temperature–mortality associations.

## Conflicts of interest statement

The authors declare that they have no conflicts of interest with regard to the content of this report.

## ACKNOWLEDGMENTS

The authors thank the anonymous referee for providing helpful comments that improved the quality of the manuscript. The authors thank Thomas Reerink for postprocessing the EC-Earth simulations and for making the data available. Additionally, the authors thank Louise Parsons Chini, George Hurtt, and Peter Lawrence for their invaluable assistance in integrating the MAgPIE land cover scenarios into the ESMs via the LUH2 format.

Multi-Country Multi-City (MCC) Collaborative Research Network:

Rosana Abrutzky, Universidad de Buenos Aires, Facultad de Ciencias Sociales, Instituto de Investigaciones Gino Germani, Argentina; Yuming Guo, Department of Epidemiology and Preventive Medicine, School of Public Health and Preventive Medicine, Monash University, Melbourne, Australia, Climate, Air Quality Research Unit, School of Public Health and Preventive Medicine, Monash University, Melbourne, Australia; Shilu Tong, National Institute of Environmental Health, Chinese Center for Disease Control and Prevention, Beijing, China, School of Public Health and Social Work, Queensland University of Technology, Brisbane, Australia; Micheline de Sousa Zanotti Stagliorio Coelho, Department of Pathology, Faculty of Medicine, University of São Paulo, São Paulo, Brazil; Paulo Hilario Nascimento Saldiva, INSPER, São Paulo, Brazil; Patricia Matus Correa, Department of Public Health, Universidad de los Andes, Santiago, Chile; Nicolás Valdés Orteg, Centro Interdisciplinario de Cambio Global, Pontificia, Universidad Católica de Chile, Santiago, Chile; Haidong Kan, Department of Environmental Health, School of Public Health, Fudan University, Shanghai, China; Samuel Osorio, Department of Environmental Health, National Institute of Public Health, Mexico; Jan Kyselý, Institute of Atmospheric Physics, Academy of Sciences of the Czech Republic, Prague, Czech Republic, Faculty of Environmental Sciences, Czech University of Life Sciences, Prague, Czech Republic; Aleš Urban, Institute of Atmospheric Physics, Academy of Sciences of the Czech Republic, Prague, Czech Republic; Hans Orru, Ene Indermitte, Department of Family Medicine and Public Health, University of Tartu, Tartu, Estonia; Jouni J. K. Jaakkola, Niilo Ryti, Center for Environmental and Respiratory Health Research (CERH), University of Oulu, Oulu, Finland, Medical Research Center Oulu (MRC Oulu), Oulu University Hospital and University of Oulu, Oulu, Finland; Mathilde Pascal, Santé Publique France, Department of Environmental and Occupational Health, French National Public Health Agency, Saint Maurice, France; Alexandra Schneider, Institute of Epidemiology, Helmholtz Zentrum München – German Research Center for Environmental Health (GmbH), Neuherberg, Germany; Veronika Huber, IBE-Chair of Epidemiology, LMU Munich, Munich, Germany, Institute of Epidemiology, Helmholtz Zentrum München – German Research Center for Environmental Health (GmbH), Neuherberg, Germany; Klea Katsouyanni, Department of Hygiene, Epidemiology and Medical Statistics, National and Kapodistrian University of Athens, Athens, Greece, Environmental Research Group, School of Public Health, Imperial College, London, United Kingdom; Antonis Analitis, Department of Hygiene, Epidemiology and Medical Statistics, National and Kapodistrian University of Athens, Athens, Greece; Hanne Krage Carlsen, School of Public Health and Community Medicine, University of Gothenburg, Gothenburg, Sweden; Fatemeh Mayvaneh, University of Münster, Institute of Landscape Ecology, Climatology Research Group, Münster, Germany; Hematollah Roradeh, Geography and Urban Planning Department, University of Mazandaran, Babolsar, Iran; Patrick Goodman, Technological University Dublin, Dublin, Ireland; Ariana Zeka, UK Health Security Agency, London, United Kingdom; Raanan Raz, Braun School of Public Health and Community Medicine, The Hebrew University of Jerusalem, Israel; Paola Michelozzi, Francesca de’Donato, Department of Epidemiology, Lazio Regional Health Service, Rome, Italy; Masahiro Hashizume, Department of Global Health Policy, Graduate School of Medicine, The University of Tokyo, Tokyo, Japan; Yoonhee Kim, Department of Global Environmental Health, Graduate School of Medicine, University of Tokyo, Tokyo, Japan; Barrak Alahmad, Department of Environmental Health, Harvard T.H. Chan School of Public Health, Harvard University, Boston, Massachusetts; Magali Hurtado Diaz, Eunice Elizabeth Félix Arellano, Department of Environmental Health, National Institute of Public Health, Cuernavaca, Morelos, Mexico; Ala Overcenco, National Agency for Public Health of the Ministry of Health, Labour and Social Protection of the Republic of Moldova, Chisinau, Republic of Moldova; Danny Houthuijs, Caroline Ameling, National Institute for Public Health and the Environment (RIVM), Centre for Sustainability and Environmental Health, Bilthoven, Netherlands; Shilpa Rao, Norwegian Institute of Public Health, Oslo, Norway; Gabriel Carrasco, Institute of Tropical Medicine “Alexander von Humboldt,” Universidad Peruana Cayetano Heredia, Lima, Peru; Xerxes Seposo, Department of Hygiene, Graduate School of Medicine, Hokkaido University, Sapporo, Japan, School of Tropical Medicine and Global Health, Nagasaki University, Nagasaki, Japan; Paul LC Chua, Department of Global Health Policy, Graduate School of Medicine, The University of Tokyo, Tokyo, Japan; Susana das Neves Pereira da Silva, Department of Epidemiology, Instituto Nacional de Saúde Dr. Ricardo Jorge, Lisbon, Portugal; Joana Madureira, Department of Epidemiology, Instituto Nacional de Saúde Dr. Ricardo Jorge, Lisbon, Portugal, EPIUnit - Instituto de Saúde Pública, Universidade do Porto, Porto, Portugal, Laboratório para a Investigação Integrativa e Translacional em Saúde Populacional (ITR), Porto, Portugal; Iulian-Horia Holobaca, Faculty of Geography, Babes-Bolay University, Cluj-Napoca, Romania; Noah Scovronick, Department of Environmental Health. Rollins School of Public Health, Emory University, Atlanta, Georgia; Fiorella Acquaotta, Department of Earth Sciences, University of Torino, Italy; Ho Kim, Graduate School of Public Health, Seoul National University, Seoul, South Korea; Whanhee Lee, School of Biomedical Convergence Engineering, College of Information and Biomedical Engineering, Pusan National University, Yangsan, South Korea, Institute of Ewha-SCL for Environmental Health (IESEH), Seoul, South Korea; Aurelio Tobias, Institute of Environmental Assessment and Water Research (IDAEA), Spanish Council for Scientific Research (CSIC), Barcelona, Spain; Carmen Íñiguez, Department of Statistics and Computational Research. Universitat de València, València, Spain, CIBERESP, Madrid, Spain; Bertil Forsberg, Department of Public Health and Clinical Medicine, Umeå University, Sweden; Martina S. Ragettli, Swiss Tropical and Public Health Institute, Allschwill, Switzerland, University of Basel, Basel, Switzerland; Yue Leon Guo, Environmental and Occupational Medicine, National Taiwan University (NTU) College of Medicine and NTU Hospital, Taipei, Taiwan, National Institute of Environmental Health Science, National Health Research Institutes, Zhunan, Taiwan, Graduate Institute of Environmental and Occupational Health Sciences, NTU College of Public Health, Taipei, Taiwan; Shih-Chun Pan, National Institute of Environmental Health Science, National Health Research Institutes, Zhunan, Taiwan; Shanshan Li, Department of Epidemiology and Preventive Medicine, School of Public Health and Preventive Medicine, Monash University, Melbourne, Australia, Climate, Air Quality Research Unit, School of Public Health and Preventive Medicine, Monash University, Melbourne, Australia; Valentina Colistro, Department of Quantitative Methods, School of Medicine, University of the Republic, Montevideo, Uruguay; Antonella Zanobetti, Joel Schwartz, Department of Environmental Health, Harvard T.H. Chan School of Public Health, Harvard University, Boston, Massachusetts; Tran Ngoc Dang, Institute of Research and Development, Duy Tan, University, Da Nang, Vietnam, Department of Environmental Health, Faculty of Public Health, Department of Environmental Health, University of Medicine and Pharmacy at Ho Chi Minh City, Ho Chi Minh City, Vietnam; Do Van Dung, Department of Environmental Health, Faculty of Public Health, Department of Environmental Health, University of Medicine and Pharmacy at Ho Chi Minh City, Ho Chi Minh City, Vietnam; and John Paul Cauchi, Queen Mary University of London, Malta Campus, Triq l-Arċisqof Pietru Pace Victoria, Malta.

## Supplementary Material
