# Supplementary material for: Impacts of land-use and land-cover changes on temperature-related mortality
Source: Environ Epidemiol. 2024 Oct 21;8(6):e337. doi: 10.1097/EE9.0000000000000337 (PMC11495778; doi:10.1097/EE9.0000000000000337)
Supplement: Supplementary file 1 [file ee9-8-e337-s001.pdf]

Supplementary Information: Impacts of land-use and land-cover changes on temperature-related mortality

Authors: Anton Orlov[1,*], Steven J. De Hertog[2,3], Felix Havermann[4], Suqi Guo[4], Iris Manola[5], Quentin Lejeune[6], Carl Schleussner[6], Wim Thiery[2], Julia Pongratz[4,7], Florian Humpenöder[8], Alexander Popp[8,9], Kristin Aunan[1], Ben Armstrong[10], Dominic Royé[11,12], Ivana Cvijanovic[13], Eric Lavigne[14,15], Souzana Achilleos[16], Michelle Bell[17,18], Pierre Masselot[19], Francesco Sera[20], Ana Maria Vicedo-Cabrera[21,22], Antonio Gasparrini[19], Multi-Country Multi-City (MCC) Collaborative Research Network[†], Malcolm N. Mistry[19,23,*]

Affiliations:

[1] CICERO Center for International Climate Research, Oslo, Norway
[2] Vrije Universiteit Brussel, Department of Water and Climate, Brussels, Belgium
[3] Universiteit Gent, Q-ForestLab, Department of Environment, Ghent, Belgium
[4] Ludwig-Maximilians-Universität in Munich, Department of Geography, Munich, Germany
[5] Vrije Universiteit Amsterdam, Institute for Environmental studies, Amsterdam, Netherlands
[6] Climate Analytics, Berlin, Germany
[7] Max Planck Institute for Meteorology, Hamburg, Germany
[8] Potsdam Institute for Climate Impact Research (PIK), Member of the Leibniz Association, Potsdam, Germany
[9] Faculty of Organic Agricultural Sciences, University of Kassel, Witzenhausen, Germany.
[10] Department of Public Health Environments and Society, London School of Hygiene & Tropical Medicine, London, United Kingdom
[11] Climate Research Foundation (FIC), Madrid, Spain
[12] CIBERESP, Madrid. Spain
[13] Barcelona Institute for Global Health, Barcelona, Spain
[14] School of Epidemiology & Public Health, Faculty of Medicine, University of Ottawa, Ottawa, Canada
[15] Environmental Health Science & Research Bureau, Health Canada, Ottawa, Canada
[16] Department of Primary Care and Population Health, University of Nicosia Medical School, Nicosia, Cyprus
[17] School of the Environment, Yale University, New Haven CT, USA
[18] School of Health Policy and Management, College of Health Sciences, Korea University, Seoul, Republic of Korea
[19] Environment & Health Modelling (EHM) Lab, Department of Public Health Environments and Society, London School of Hygiene & Tropical Medicine, London, United Kingdom
[20] Department of Statistics, Computer Science and Applications "G. Parenti", University of Florence, Florence, Italy
[21] Institute of Social and Preventive Medicine, University of Bern, Bern, Switzerland
[22] Oeschger Center for Climate Change Research, University of Bern, Bern, Switzerland
[23] Department of Economics, Ca' Foscari University of Venice, Italy

*** Corresponding authors:**

Anton Orlov (anton.orlov@cicero.oslo.no, +4722004733, Postboks 1129 Blindern, 0318 Oslo, Norway)

Malcolm N. Mistry (malcolm.mistry@lshtm.ac.uk, +44(0)2076368636, 15-17 Tavistock Place, London WC1H 9SH, UK)

† Multi-Country Multi-City (MCC) Collaborative Research Network:

Rosana Abrutzky[24], Yuming Guo[25,26], Shilu Tong[27,28], Micheline de Sousa Zanotti Stagliorio Coelho[29], Paulo Hilario Nascimento Saldiva[30], Patricia Matus Correa[31], Nicolás Valdés Orteg[32], Haidong Kan[33], Samuel Osorio[34], Jan Kyselý[35,36], Aleš Urban[35], Hans Orru[37], Ene Indermitte[37], Jouni J. K. Jaakkola[38,39], Niilo Ryti[38,39], Mathilde Pascal[40], Alexandra Schneider[41], Veronika Huber[41,42], Klea Katsouyanni[43,44], Antonis Analitis[43], Hanne Krage Carlsen[45], Fatemeh Mayvaneh[46], Hematollah Roradeh[47], Patrick Goodman[48], Ariana Zeka[49], Raanan Raz[50], Paola Michelozzi[51], Francesca de'Donato[51], Masahiro Hashizume[52], Yoonhee Kim[53], Barrak Alahmad[54], Magali Hurtado Diaz[55], Eunice Elizabeth Félix Arellano[55], Ala Overcenco[56], Danny Houthuijs[57], Caroline Ameling[57], Shilpa Rao[58], Gabriel Carrasco[59], Xerxes Seposo[60,61], Paul LC Chua[52], Susana das Neves Pereira da Silva[62], Joana Madureira[62,63,64], Iulian-Horia Holobaca[65], Noah Scovronick[66], Fiorella Acquaotta[67], Ho Kim[68], Whanhee Lee[69,70], Aurelio Tobias[71], Carmen Íñiguez[72,12], Bertil Forsberg[73], Martina S. Ragettli[74,75], Yue Leon Guo[76,77,78], Shih-Chun Pan[77], Shanshan Li[25,26], Valentina Colistro[79], Antonella Zanobetti[54], Joel Schwartz[54], Tran Ngoc Dang[80,81], Do Van Dung[81] and John Paul Cauchi[82].

[24] Universidad de Buenos Aires, Facultad de Ciencias Sociales, Instituto de Investigaciones Gino Germani, Argentina
[25] Department of Epidemiology and Preventive Medicine, School of Public Health and Preventive Medicine, Monash University, Melbourne, Australia
[26] Climate, Air Quality Research Unit, School of Public Health and Preventive Medicine, Monash University, Melbourne, Australia
[27] National Institute of Environmental Health, Chinese Center for Disease Control and Prevention, Beijing, China
[28] School of Public Health and Social Work, Queensland University of Technology, Brisbane, Australia
[29] Department of Pathology, Faculty of Medicine, University of São Paulo, São Paulo, Brazil
[30] INSPER, São Paulo, Brazil
[31] Department of Public Health, Universidad de los Andes, Santiago, Chile
[32] Centro Interdisciplinario de Cambio Global, Pontificia, Universidad Católica de Chile, Santiago, Chile
[33] Department of Environmental Health, School of Public Health, Fudan University, Shanghai, China
[34] Department of Environmental Health, National Institute of Public Health, Mexico
[35] Institute of Atmospheric Physics, Academy of Sciences of the Czech Republic, Prague, Czech Republic
[36] Faculty of Environmental Sciences, Czech University of Life Sciences, Prague, Czech Republic.
[37] Department of Family Medicine and Public Health, University of Tartu, Tartu, Estonia
[38] Center for Environmental and Respiratory Health Research (CERH), University of Oulu, Oulu, Finland
[39] Medical Research Center Oulu (MRC Oulu), Oulu University Hospital and University of Oulu, Oulu, Finland
[40] Santé Publique France, Department of Environmental and Occupational Health, French National Public Health Agency, Saint Maurice, France
[41] Institute of Epidemiology, Helmholtz Zentrum München – German Research Center for Environmental Health (GmbH), Neuherberg, Germany
[42] IBE-Chair of Epidemiology, LMU Munich, Munich, Germany
[43] Department of Hygiene, Epidemiology and Medical Statistics, National and Kapodistrian University of Athens, Greece
[44] Environmental Research Group, School of Public Health, Imperial College, London, UK
[45] School of Public Health and Community Medicine, University of Gothenburg, Gothenburg, Sweden
[46] University of Münster, Institute of Landscape Ecology, Climatology Research Group, Münster, Germany
[47] Geography and Urban Planning Department, University of Mazandaran, Babolsar, Iran
[48] Technological University Dublin, Ireland
[49] UK Health Security Agency, London, UK
[50] Braun School of Public Health and Community Medicine, The Hebrew University of Jerusalem, Israel
[51] Department of Epidemiology, Lazio Regional Health Service, Rome, Italy
[52] Department of Global Health Policy, Graduate School of Medicine, The University of Tokyo, Tokyo, Japan
[53] Department of Global Environmental Health, Graduate School of Medicine, University of Tokyo, Tokyo, Japan
[54] Department of Environmental Health, Harvard T.H. Chan School of Public Health, Harvard University, Boston, MA, USA
[55] Department of Environmental Health, National Institute of Public Health, Cuernavaca, Morelos, Mexico
[56] National Agency for Public Health of the Ministry of Health, Labour and Social Protection of the Republic of Moldova

[57] National Institute for Public Health and the Environment (RIVM), Centre for Sustainability and Environmental Health, Bilthoven, Netherlands
[58] Norwegian institute of Public Health, Oslo, Norway
[59] Institute of Tropical Medicine "Alexander von Humboldt", Universidad Peruana Cayetano Heredia, Lima, Peru
[60] Department of Hygiene, Graduate School of Medicine, Hokkaido University, Sapporo, Japan
[61] School of Tropical Medicine and Global Health, Nagasaki University, Nagasaki, Japan
[62] Department of Epidemiology, Instituto Nacional de Saúde Dr. Ricardo Jorge, Lisbon, Portugal
[63] EPIUnit - Instituto de Saúde Pública, Universidade do Porto, Porto, Portugal
[64] Laboratório para a Investigação Integrativa e Translacional em Saúde Populacional (ITR), Porto, Portugal
[65] Faculty of Geography, Babes-Bolay University, Cluj-Napoca, Romania
[66] Department of Environmental Health. Rollins School of Public Health, Emory University, Atlanta, USA
[67] Department of Earth Sciences, University of Torino, Italy
[68] Graduate School of Public Health, Seoul National University, Seoul, South Korea
[69] School of Biomedical Convergence Engineering, College of Information and Biomedical Engineering, Pusan National University, Yangsan, South Korea
[70] Institute of Ewha-SCL for Environmental Health (IESEH)
[71] Institute of Environmental Assessment and Water Research (IDAEA), Spanish Council for Scientific Research (CSIC), Barcelona, Spain
[72] Department of Statistics and Computational Research. Universitat de València, València, Spain
[73] Department of Public Health and Clinical Medicine, Umeå University, Sweden
[74] Swiss Tropical and Public Health Institute, Allschwill, Switzerland
[75] University of Basel, Basel
[76] Environmental and Occupational Medicine, National Taiwan University (NTU) College of Medicine and NTU Hospital, Taipei, Taiwan
[77] National Institute of Environmental Health Science, National Health Research Institutes, Zhunan, Taiwan
[78] Graduate Institute of Environmental and Occupational Health Sciences, NTU College of Public Health, Taipei, Taiwan
[79] Department of Quantitative Methods, School of Medicine, University of the Republic, Montevideo, Uruguay
[80] Institute of Research and Development, Duy Tan, University, Da Nang, Vietnam
[81] Department of Environmental Health, Faculty of Public Health, Department of Environmental Health, University of Medicine and Pharmacy at Ho Chi Minh City, Ho Chi Minh City, Vietnam

[82] Queen Mary University of London, Malta Campus, Triq l-Arċisqof Pietru Pace Victoria, Malta

Contents:

(1) **Description of the two-stage empirical framework**

(2) **Computation of excess mortality (EM)**

(3) **Projection and quantification of the temperature-related EM**

(4) **List of Figures:**

Figure S1: Land cover patterns showing the amount of cropland and forest in the control simulations and the change for the Sustainability and Inequality scenarios by the end of the century.

Figure S2: Change in mid-century average near-surface temperature under noLULCC scenario relative to historical.

Figure S3: Change in end-century average near-surface temperature under noLULCC scenario relative to historical.

Figure S4: Change in mid-century average near-surface temperature under Sustainability scenario relative to noLULCC scenario.

Figure S5: Change in end-century average near-surface temperature under Sustainability scenario relative to noLULCC scenario.

Figure S6: Change in mid-century average near-surface temperature under Inequality scenario relative to noLULCC scenario.

Figure S7: Change in end-century average near-surface temperature under Inequality scenario relative to noLULCC scenario.

Figure S8: Change in mid-century fraction of all-cause excess mortality (due to cold, heat and total) under noLULCC scenario relative to historical.

Figure S9: Change in end-century fraction of all-cause excess mortality (due to cold, heat and total) under noLULCC scenario relative to historical.

Figure S10: Change in mid-century fraction of all-cause excess mortality (due to cold, heat and total) under Sustainability scenario relative to the noLULCC scenario.

Figure S11: Change in end-century fraction of all-cause excess mortality (due to cold, heat and total) under Sustainability scenario relative to the noLULCC scenario.

Figure S12: Change in mid-century fraction of all-cause excess mortality (due to cold, heat and total) under Inequality scenario relative to the noLULCC scenario.

Figure S13: Change in end-century fraction of all-cause excess mortality (due to cold, heat and total) under Inequality scenario relative to the noLULCC scenario.

Figure S14 Regionally aggregated change in mid-century fraction of all-cause excess mortality.

Figure S15: Regionally aggregated change in end-century fraction of all-cause excess mortality.

Figure S16: Mid-century regionally aggregated multi-model mean change in the fraction of excess temperature-related mortality.

Figure S17: End-century regionally aggregated multi-model mean change in the fraction of excess temperature-related mortality.

Figure S18: Overall cumulative exposure-response associations (relative risk) for representative big cities using MCC data.

Figure S19: Schematic illustration of a shift in mid- and end-century temperature distribution under noLULCC scenario relative to historical, and the historical exposure-response association (relative risk) for Santiago (Chile)

Figure S20: Schematic illustration of a shift in end-century temperature distribution under Inequality and Sustainability scenarios relative to noLULCC, and the historical exposure-response association (relative risk) for Asuncion (Paraguay)

(5) List of Tables:

Table S1: MCC weather station and mortality data by countries.

Table S2: Detailed summary statistics (File Supplementary_Table_S2.xlsx).

- Excess mortality and fraction of excess mortality (%) – City level, by ESM, scenario
- Excess mortality and fraction of excess mortality (%) – Country level, by ESM, scenario
- Excess mortality and fraction of excess mortality (%) – Regional level, by ESM, scenario
- MCC Total mortality – Countries
- MCC Total mortality – Regions

- MCC Total mortality – Global
- Number of MCC cities by countries
- Length of mortality time series by countries (including start and end year)
- Summary statistics of MCC mortality, temperature - City level.
    - Counts of all-cause or non-external daily mortality with % of missing data
    - Average daily mean temperature [Units: ° C]
    - Median daily mean temperature including Inter-Quartile Range (IQR) [Units: ° C]
    - Minimum Mortality Temperature (MMT) and Percentile (MMP) [Units: ° C]

Table S3: Changes in global mean temperature by ESM and scenario.

(1) Two-stage Empirical Framework

We estimated the location-specific historical temperature-mortality associations using the well-established two-stage time-series analysis. We applied a quasi-Poisson regression with distributed lag non-linear models (DLNM) and multivariate random meta-regression using data from the Multi-Country Multi-City (MCC) Collaborative Research Network (http://mccstudy.lshtm.ac.uk/) (described in main text and in Table S1 here, and in earlier studies: Gasparrini, 2014; Gasparrini et al., 2015, 2017).

The first stage of the two-stage framework involves independent estimation of the location-specific association between daily observed outdoor air temperature ($T_{obs,}$ Units: °C) and daily mortality counts (D_{obs}) using a quasi-Poisson regression model with a cross-basis for temperature and by controlling for confounders (i.e., seasonal and long-term trends, and day of the week) (Equation 1).

$$\log[E(D_{obs})] = \alpha + s(T_{obs};\, \theta) + f(t;\, \beta) + I(w;\, \gamma) \qquad \text{Eq. (1)}$$

where $\log[E(D_{obs})]$ denotes the natural logarithm of expected value of daily mortality counts.

The cross-basis which is represented by $s(T_{obs};\, \theta)$, was specified as a quadratic B-spline function with three internal knots at the 10th, 75th and 90th percentiles of the location-specific observed temperature distribution to model the exposure-response curve, and a natural cubic B-spline function with three internal knots at equally spaced values in the log scale over 21 days of lag for the lag-response dimension. The seasonal and long-term trends were controlled with a natural cubic spline function f with 8 degrees of freedom per year. Similarly, the differences due to the day of the week *(w; a categorical variable)* were accounted for by using an indicator function I. Cross-basis coefficients $\boldsymbol{\theta}$ are then reduced to represent the overall cumulative (over lags) exposure-response function (Gasparrini and Armstrong, 2013).

In the second stage, reduced coefficients $\boldsymbol{\theta}$ were pooled into a multi-variate multi-level meta-regression model. To account for heterogeneity across the locations, meta-predictors included climate type (Koppen-Geiger classification), country-level GDP, and an average and range of mean daily temperature across the whole period. City and country were added as random effects to capture any structural differences not represented by the above meta-predictors. The meta-regression analysis allows the locations with less stable results to utilise the information (heterogenous effects) from other locations with similar characteristics (Gasparrini et al., 2012; θ_b thus providing improved estimates of temperature-mortality associations at location level, defined as best linear unbiased predictions (BLUPs) . Put differently, BLUPs borrow information across units within the same hierarchical level and can offer more accurate estimates, especially in locations with small daily mortality counts or short series. This approach enables more robust estimates of relative-risk or risk ratios (RRs) in individual locations compared to location-specific models (Gasparrini and Leone, 2014; Gasparrini et al., 2015). RR and excess mortality are computed as post-processing routing after condensing a two-stage time-series analysis.

The resulting overall cumulative exposure-response associations (RR) for selected cities representative of the 52 countries are shown in Figure S17. The RR is interpreted as the relative increase in risk of mortality associated with a

certain temperature versus a reference value. The reference temperature, chosen as the temperature corresponding to minimum mortality risk (MMT) is interpreted as the optimal temperature for that specific location. The MMT can therefore be considered as the counterfactual condition for the definition of the excess mortality discussed below. Finally, the uncertainty in the estimation of the exposure-response relationships is quantified using 1000 Monte Carlo simulations to obtain 95% empirical CIs (eCIs) (Gasparrini and Leone, 2014), shown as shaded grey bands in Figures S17.

(2) Computation of excess mortality (EM)

Using the exposure-response associations estimated at each location, we next quantify the health impacts by way of excess mortality (EM; also referred to as attributable numbers; AN) from cold, heat, and net (cold + heat) (Gasparrini 2014; Gasparrini and Leone, 2014; Gasparrini et al., 2015; Vicedo-Cabrera et al., 2019). The EM or AN can be interpreted as the excess deaths due to non-optimal temperature when compared to a hypothetical situation in which temperature is constantly equal to the MMT. As noted previously by Honda et al., 2014 and Gasparrini and Leone, 2014, a null exposure condition cannot be defined for studies employing temperature as an environmental exposure variable, and a reasonable choice therefore is to centre the cross-basis in Eq. (1) to the temperature of minimum risk (i.e., MMT).

Specifically, the number of deaths attributable to non-optimum temperature based on the estimated risk and the level of exposure on that specific day is computed for each day of the series. The location-specific EM (or AN) are then expressed in terms of attributable fraction (AF) computed as the ratio with the corresponding total number of cases (D_{obs}). Mathematically, daily AF and AN can be written as:

$$AF_x = 1 - \exp(-\beta_x) \qquad \text{Eq. (2)}$$

$$AN_x = D_{obs} \cdot AF_x \qquad \text{Eq. (3)}$$

where x represents a given exposure (here T_{obs}), the parameter β_x represents the risk associated with the exposure (here either cold, heat or net) corresponding to the natural logarithm of RR obtained from the regression model, and D_{obs} refers to the total number of deaths on the specific day as defined earlier (see Gasparrini and Leone, 2014 for further details). The total AN (or EM) due to temperature is the sum of AN_x for all the days in the time series as desired (e.g., weekly, monthly, annual etc.). Mortality attributable to cold and heat are computed by summing the subsets of AN from days with temperature lower or higher than the location-specific MMT. Similarly, the ratio of total AN with the total number of deaths provides the net (total) AF (or net EM), and their corresponding cumulative cold- and heat-related AF are computed by using the equivalent subsets.

In line with earlier projections-based studies (e.g., Gasparrini et al., 2017), the daily AN from cold, heat and net in our study are aggregated over each decade across 1980-2099, referred to as the estimated decadal excess (or attributable) deaths from cold, heat and net. The location-specific excess-deaths are similarly aggregated geographically to wider regions such as countries and regions, thus providing an estimate of country- or regional-scale burden of temperature-related mortality. Considering the contrast in the number of locations across countries and regions that are currently available in the MCC database, it is important to emphasise that such geographically aggregated numbers may not always be fully representative of national- or regional-scale burden of the environmental exposure being examined.

Since projection studies generally present impacts as comparative measures between climate change scenarios and/or timeframes relative to historical present-day climate, presenting results as AN (or AF) facilitates comparing differences between present-day and future AN (or AF), or between two pairs of future scenarios. In our study, we focused on comparison of AF as: (i) noLULCC scenario – historical, and (ii) LULCC scenarios (Inequality or Sustainability) – noLULCC.

(3) Projection and quantification of the temperature-related EM

We followed the approach adopted by earlier studies (Gasparrini et al., 2017; Vicedo-Cabrera et al., 2019) that involves four steps:

(a) Preparation of Earth System Model (ESM) and scenario-specific daily temperature series (T_{mod}) for each location in the study.
(b) Assembling a synthetic daily mortality time series or baseline mortality, covering the full length of the projections period.
(c) Calibration of the T_{mod} with T_{obs}.
(d) Computing relative changes in future excess-mortality.

For (a), the ESM-scenario-specific daily temperature series T_{mod} for each of the 823 locations in the period 1980-2099 were extracted by linking the coordinates of the locations with the corresponding grid cells of the three ESMs that are at 0.5° x 0.5° spatial resolution (approx. 55km x 55km at the equator).

As next step (b), the projected mortality series was assembled following an approach used in earlier studies (Gasparrini et al., 2017; Vicedo-Cabrera et al., 2019). It must be noted that while the temperature response resulting from the LULCC is driven by changes in demographic and population through SSP-driven changes in food demand, the mortality burden from non-optimal temperature is projected under the assumption of stable populations, thus ignoring demographic changes. For this reason, the projected series of mortality counts D_{mod} was assembled as the average for each day of the year from D_{obs} , and then repeated along the full projection period of T_{mod} (see Vicedo-Cabrera et al., 2019 for further details).

For step (c) involving calibration of ESMs' temperature time series, the T_{mod} from the three ESMs and the three scenarios (noLULCC, Inequality and Sustainability) were bias-adjusted using the trend-preserving bias-correction method (Hempel et al., 2013) applied in the earlier studies (Gasparrini et al., 2017; Vicedo-Cabrera et al., 2019). Briefly, this approach produces $T^{\#}_{mod}$, a series recalibrated using the monthly mean and the daily variability around the monthly mean of T_{obs}.

Lastly, for step (d), the number of deaths attributed to temperature in the future (AN_{fut}) were computed for each city i , ESM j , scenario k and day t using the standard formula:

$$AN_{fut(ijkt)} = D_{mod(it)} \cdot \frac{\left[RR_i\left(T^{\#}_{mod(ijkt)}\right)-1\right]}{\left[RR_i\left(T^{\#}_{mod(ijkt)}\right)\right]} \qquad \text{Eq. 4}$$

where $RR_i\left(T^{\#}_{mod(ijkt)}\right)$ is the relative risk associated with the projected temperature $T^{\#}_{mod}$ for city i , GCM j and day t , which is extracted from city $i-$ specific exposure-response functions, and $D_{mod(it)}$ is the projected number of deaths for city i and day t .

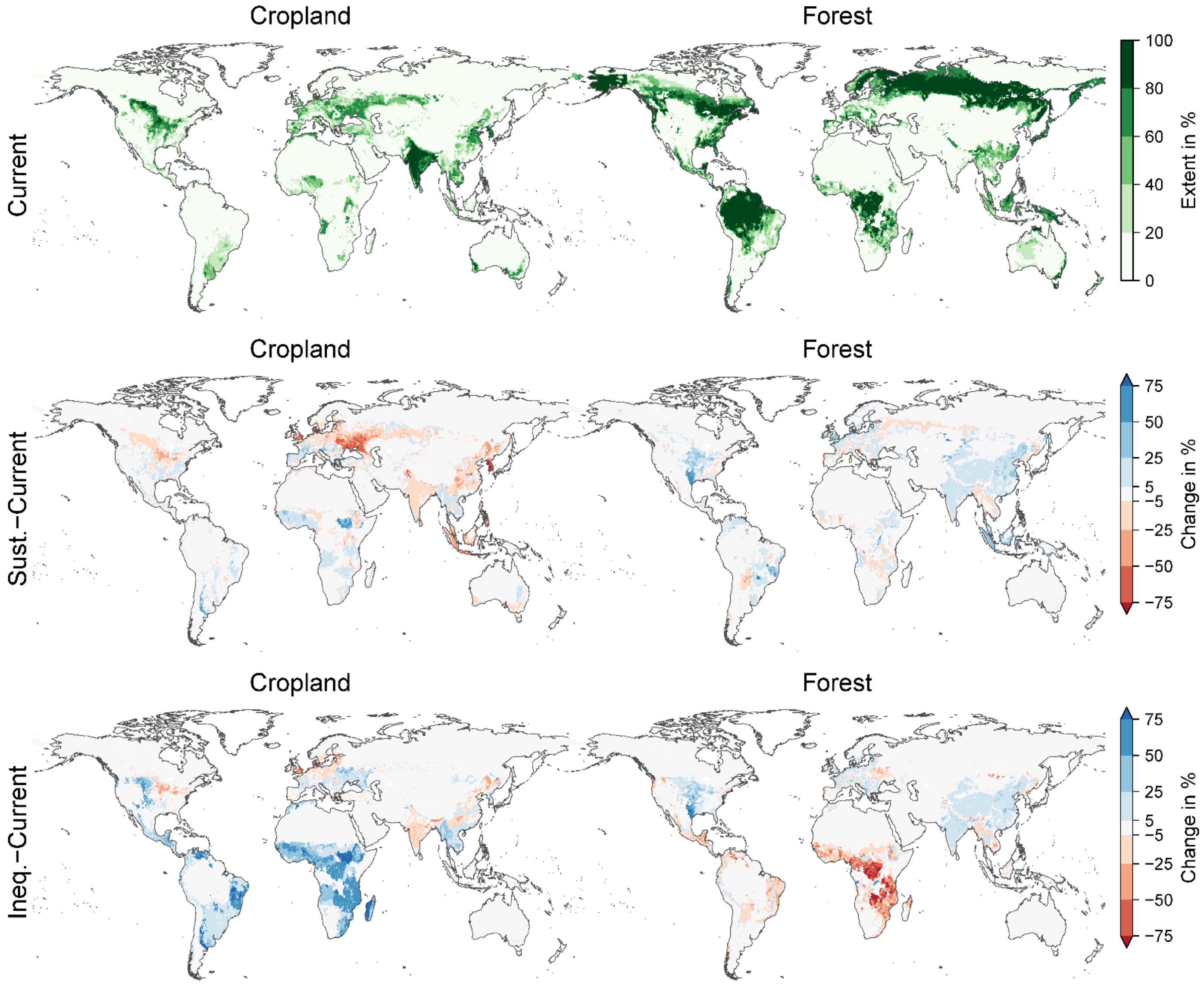

Figure S1: Land cover patterns showing the amount of cropland and forest as a grid cell fraction (%) in the control simulations (Current) (2014 land cover extent). The change for the Sustainability and Inequality scenarios (Sust.-Current and Ineq.-Current) are shown for cropland and forest by the end of the century.

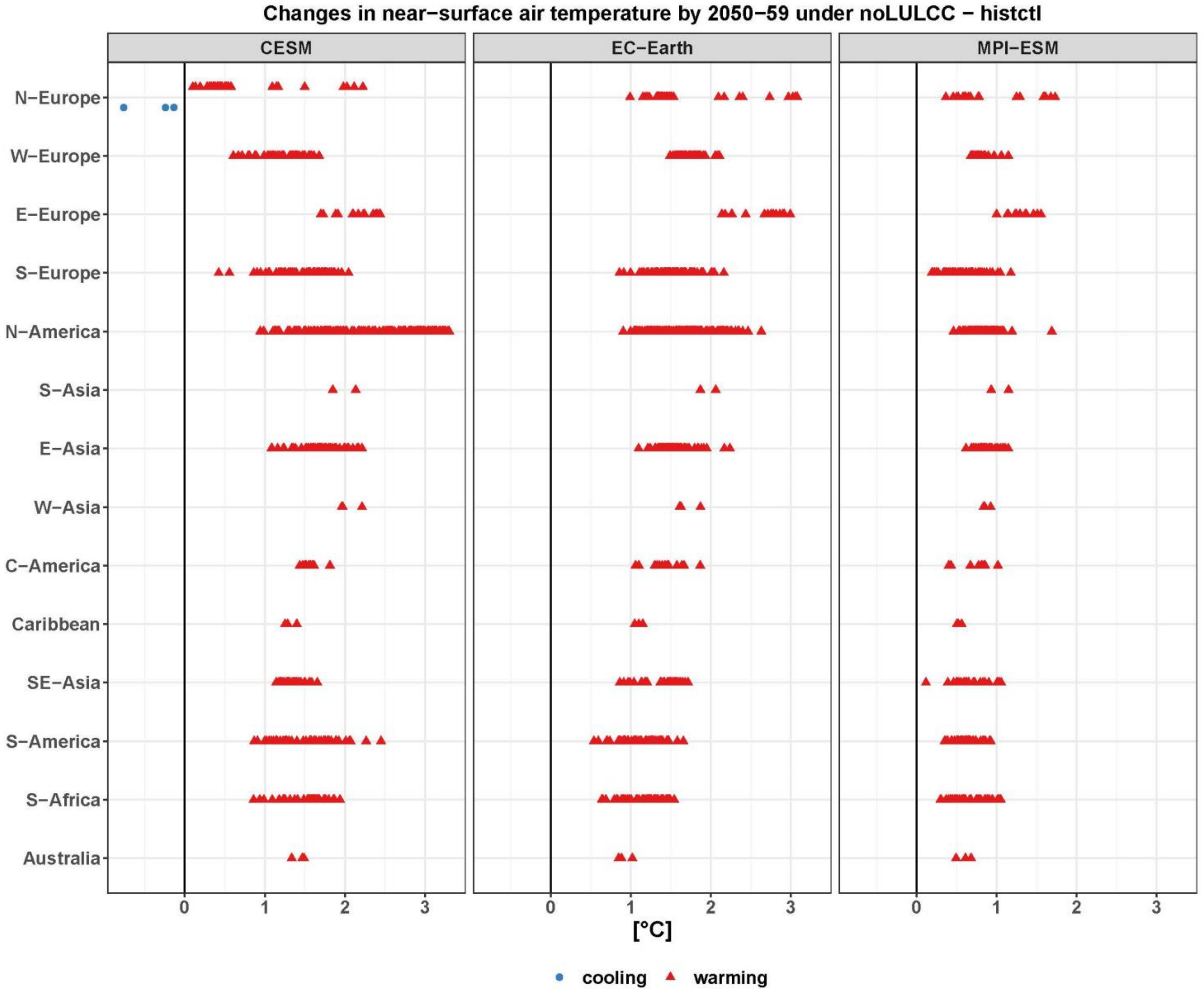

Figure S2: Change in average near-surface temperature (°C) under noLULCC scenario by 2050-2059 relative to 1980-1989. The blue circles and red triangles show cooling and warming, respectively at each MCC location by country or territory in the 14 geographic regions used in the study.

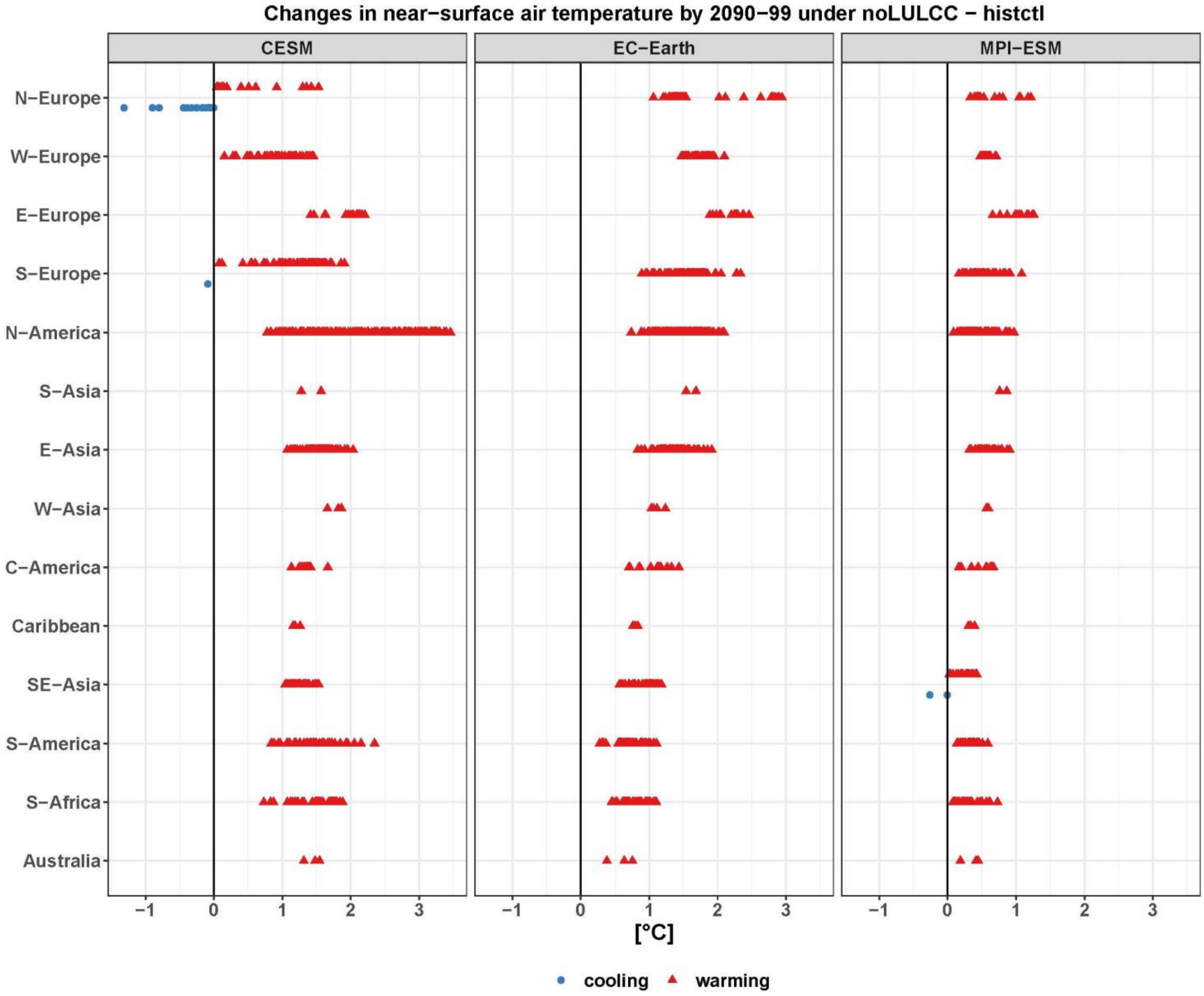

Figure S3: Same as Figure S2 but for 2090-2099 relative to 1980-1989.

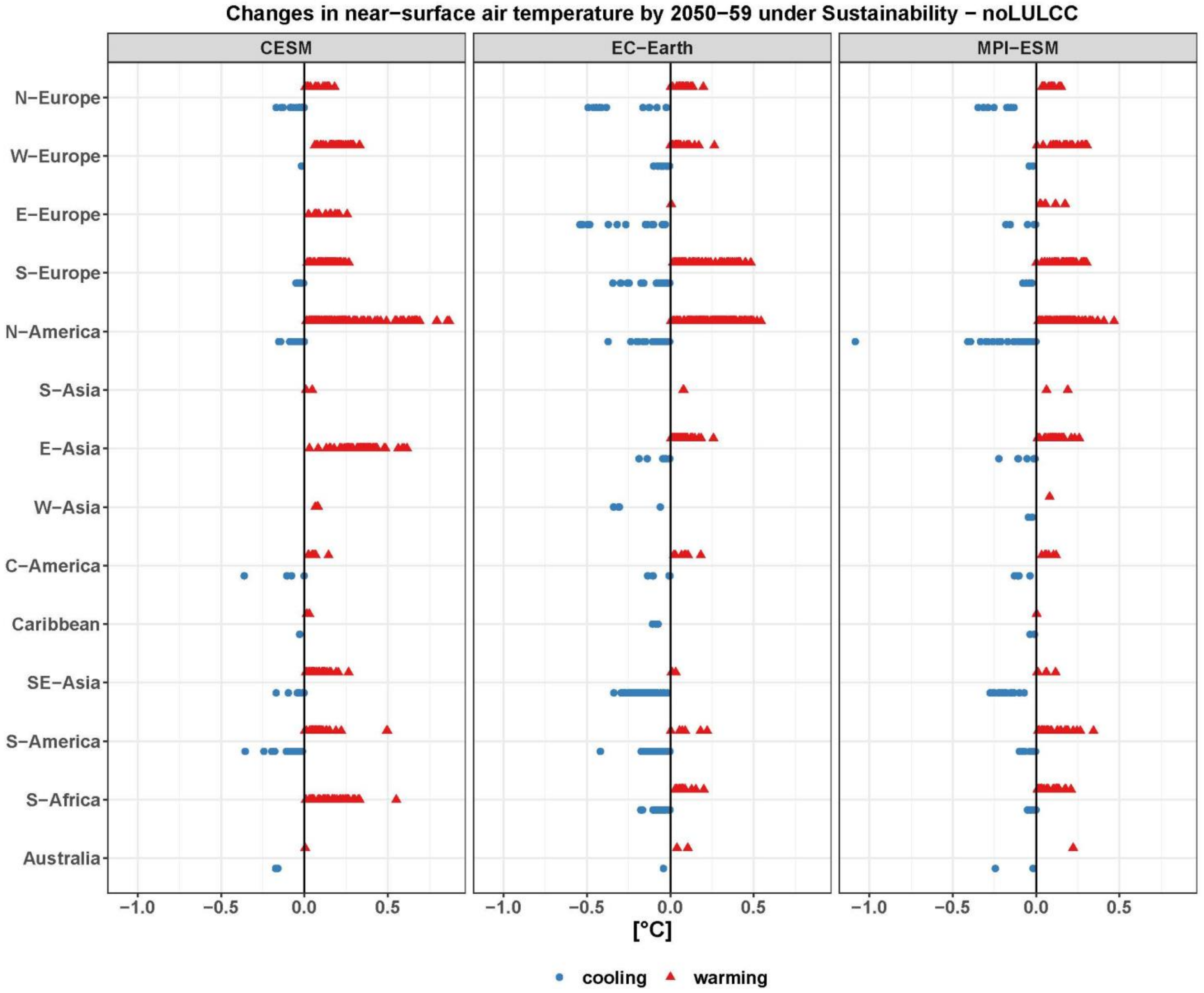

Figure S4: Change in average near-surface temperature (°C) under Sustainability scenario by 2050-2059 relative to the noLULCC scenario. The blue circles and red triangles show cooling and warming, respectively at each MCC location by country or territory in the 14 geographic regions used in the study.

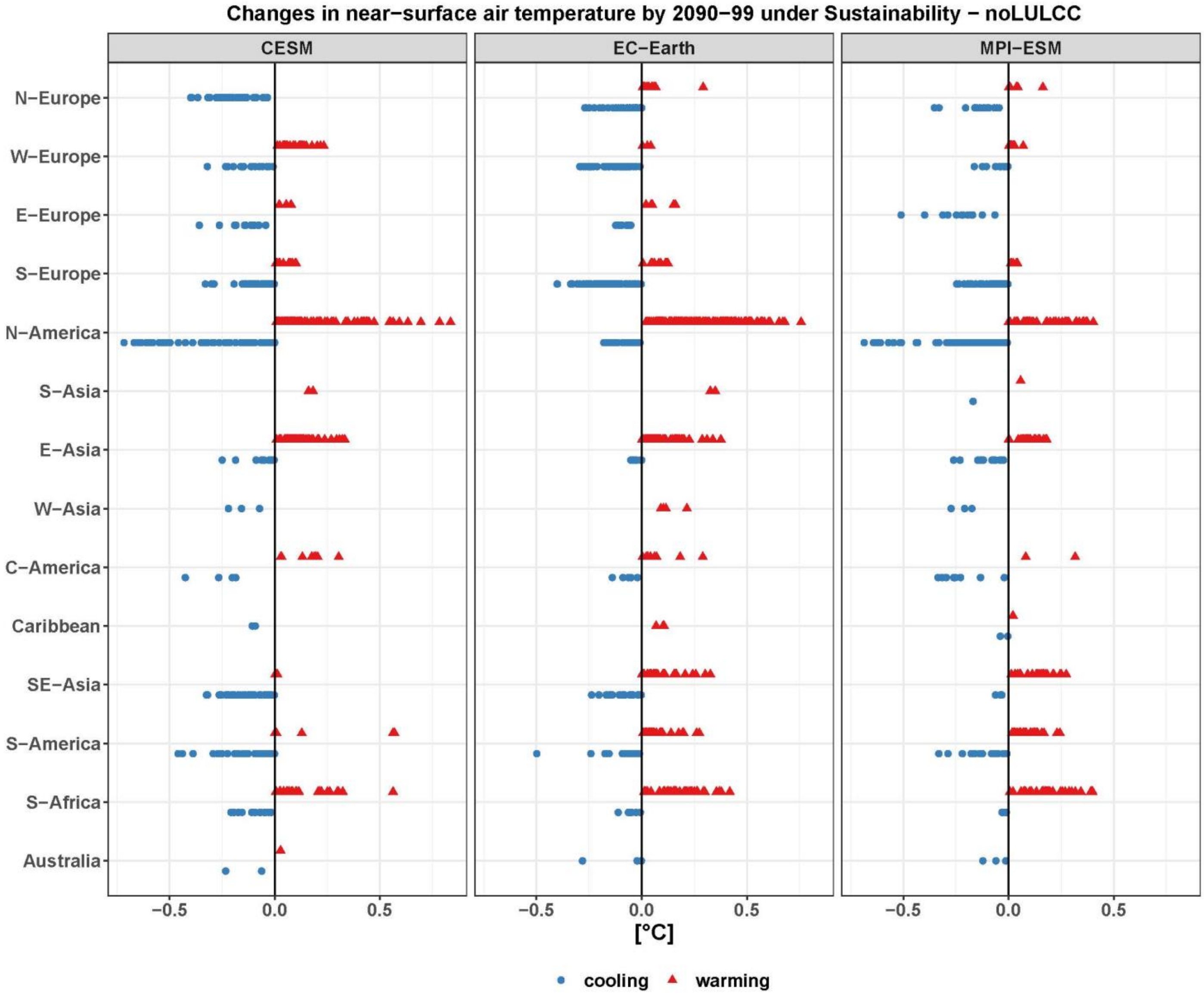

Figure S5: Same as Figure S4 but for 2090-2099.

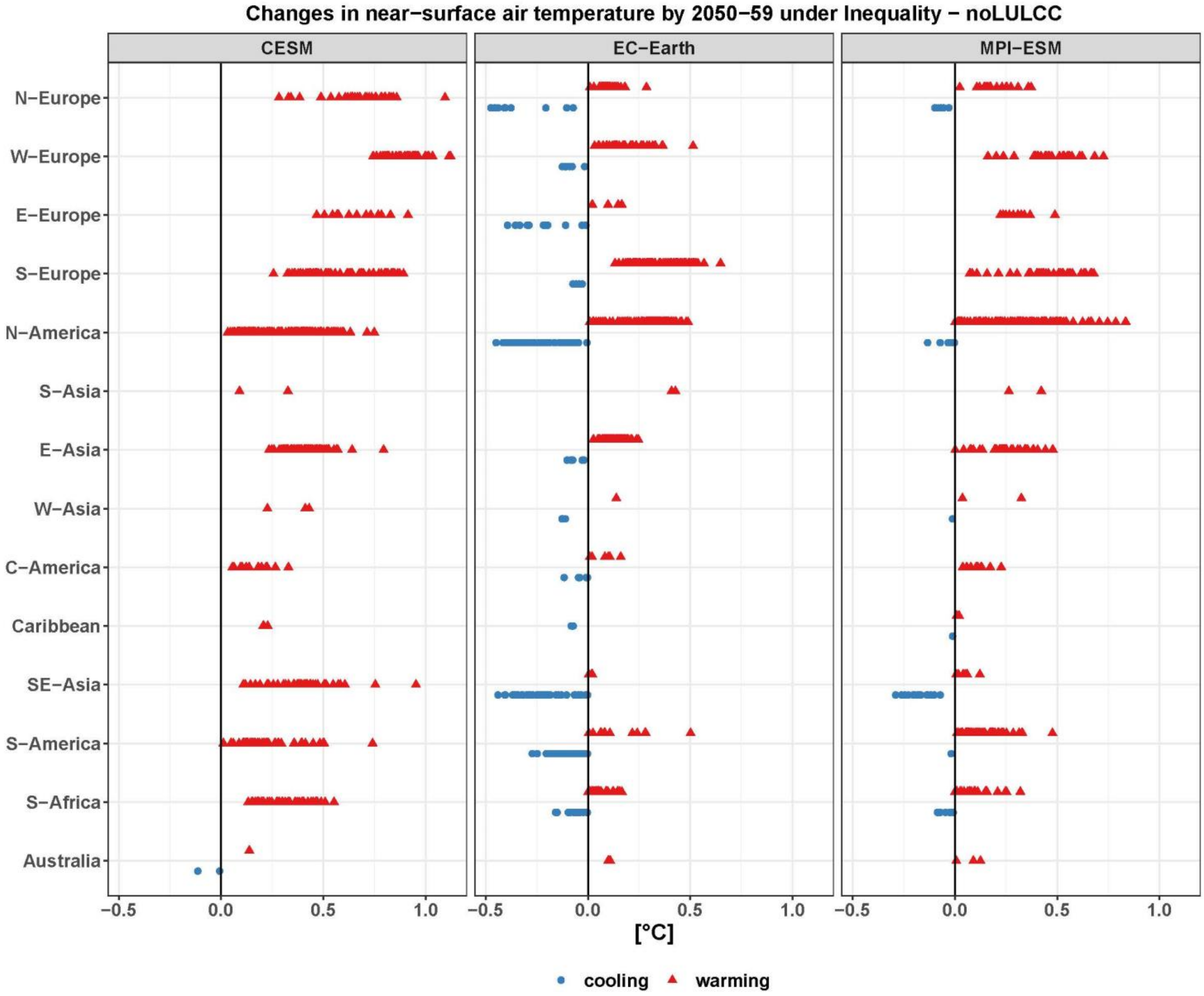

Figure S6: Change in average near-surface temperature (°C) under Inequality scenario by 2050-2059 relative to the noLULCC scenario. The blue circles and red triangles show cooling and warming, respectively at each MCC location by country or territory in the 14 geographic regions used in the study.

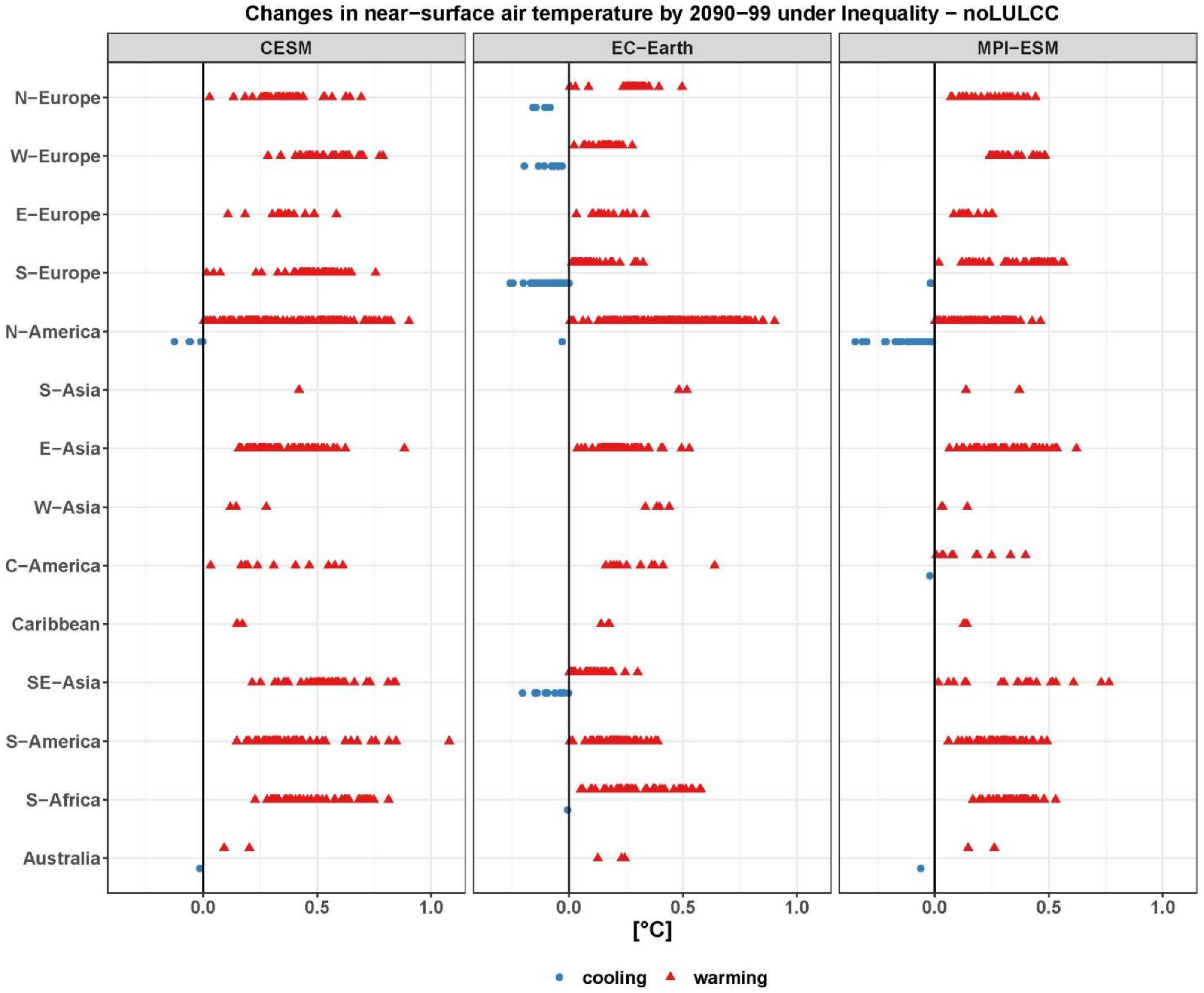

Figure S7: Same as Figure S6 but for 2090-2099.

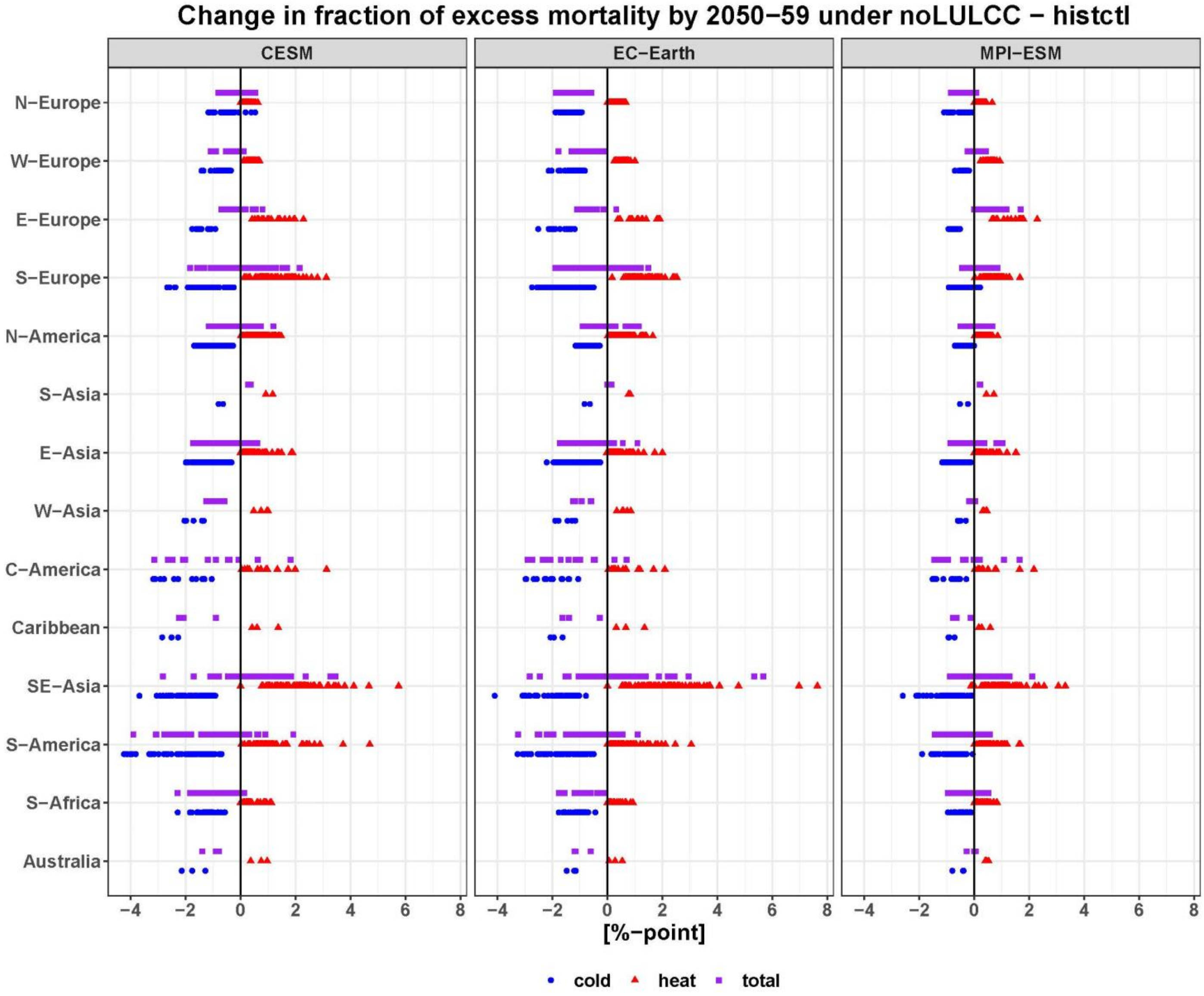

Figure S8: Change in fraction of all-cause excess cumulative mortality (in %-points) under the noLULCC scenario by 2050-2059 relative to 1980-1989. The blue circles, red triangles, and purple rectangles represent the location-specific cold, heat, and total excess mortality (expressed as fraction) respectively grouped by the countries or territories in the 14 geographic regions.

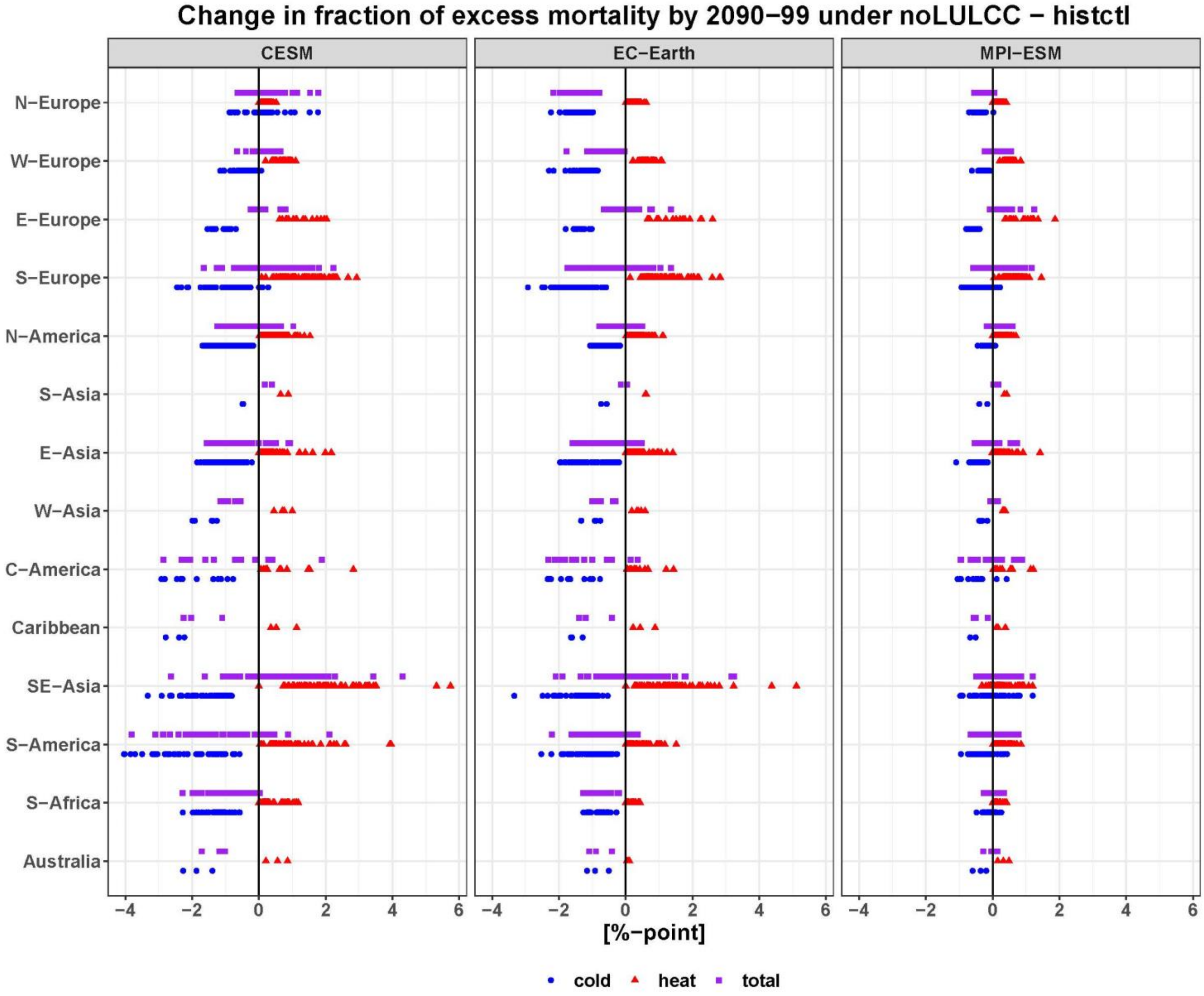

Figure S9: Same as Figure S8 but for 2090-2099 relative to 1980-1989.

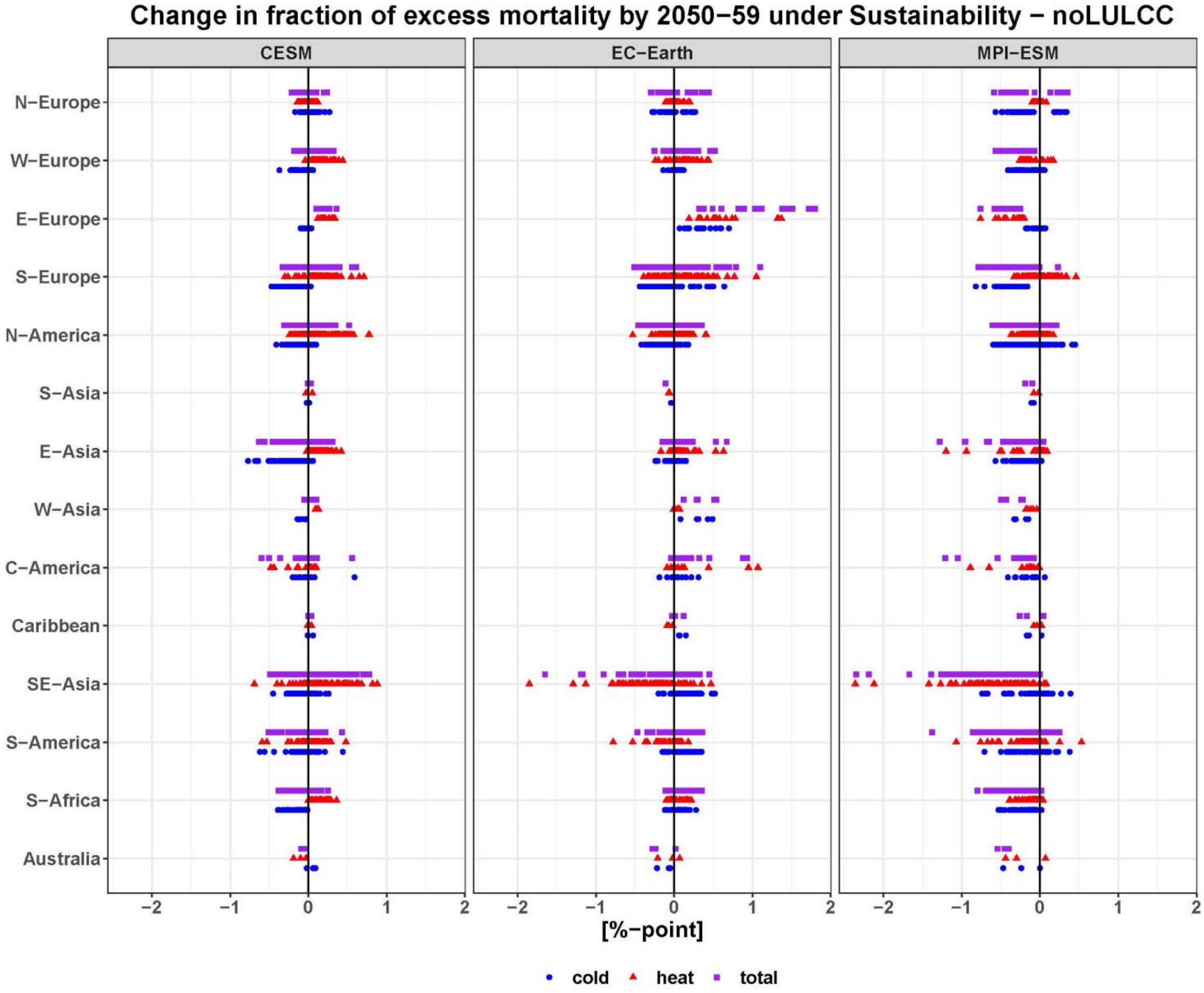

Figure S10: Change in fraction of all-cause excess cumulative mortality (in %-points) under the Sustainability scenario by 2050-2059 relative to noLULCC. The blue circles, red triangles, and purple rectangles represent the location-specific cold, heat, and total excess mortality (expressed as fraction) respectively grouped by the countries or territories in the 14 geographic regions.

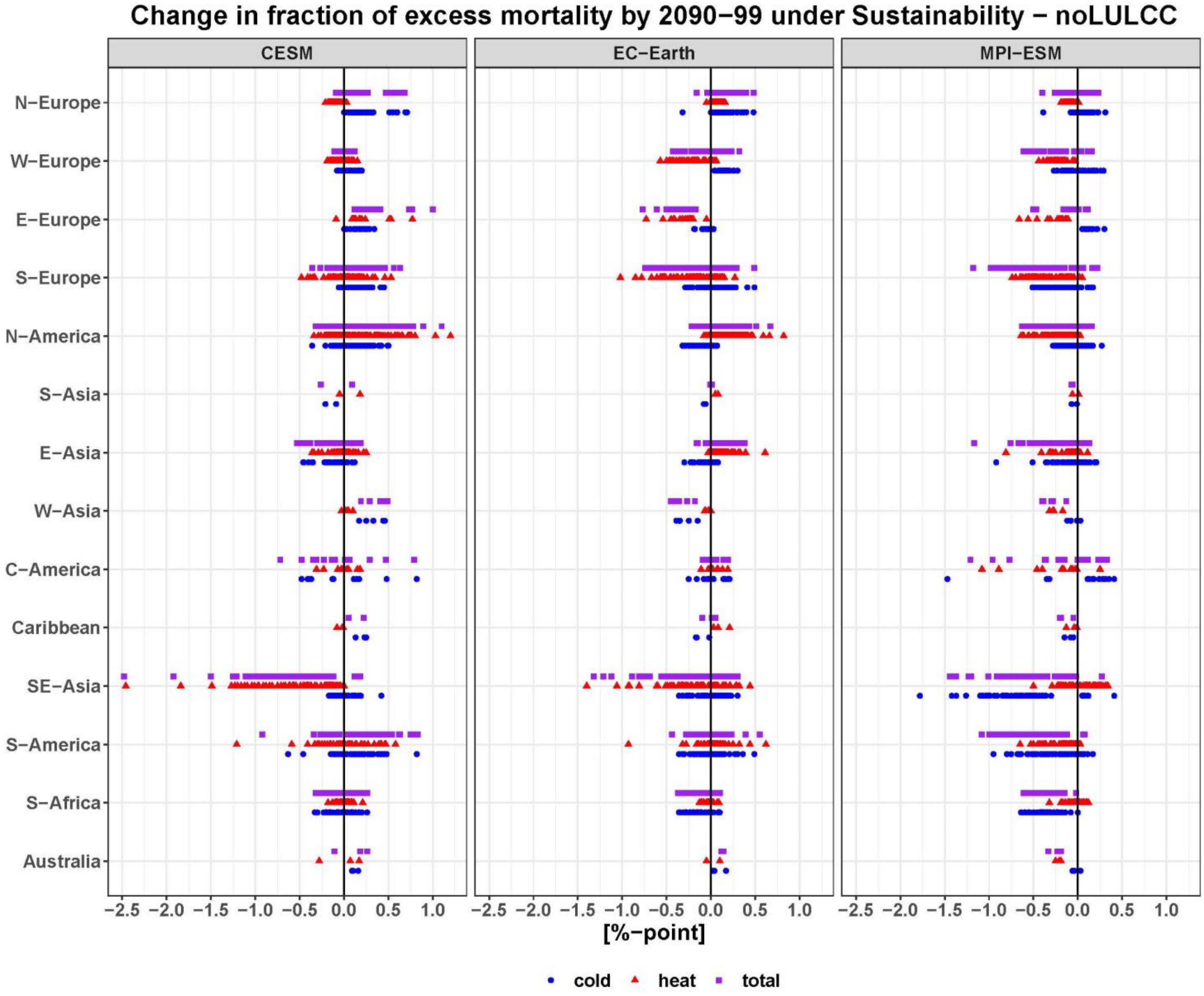

Figure S11: Same as Figure S10 but for 2090-2099.

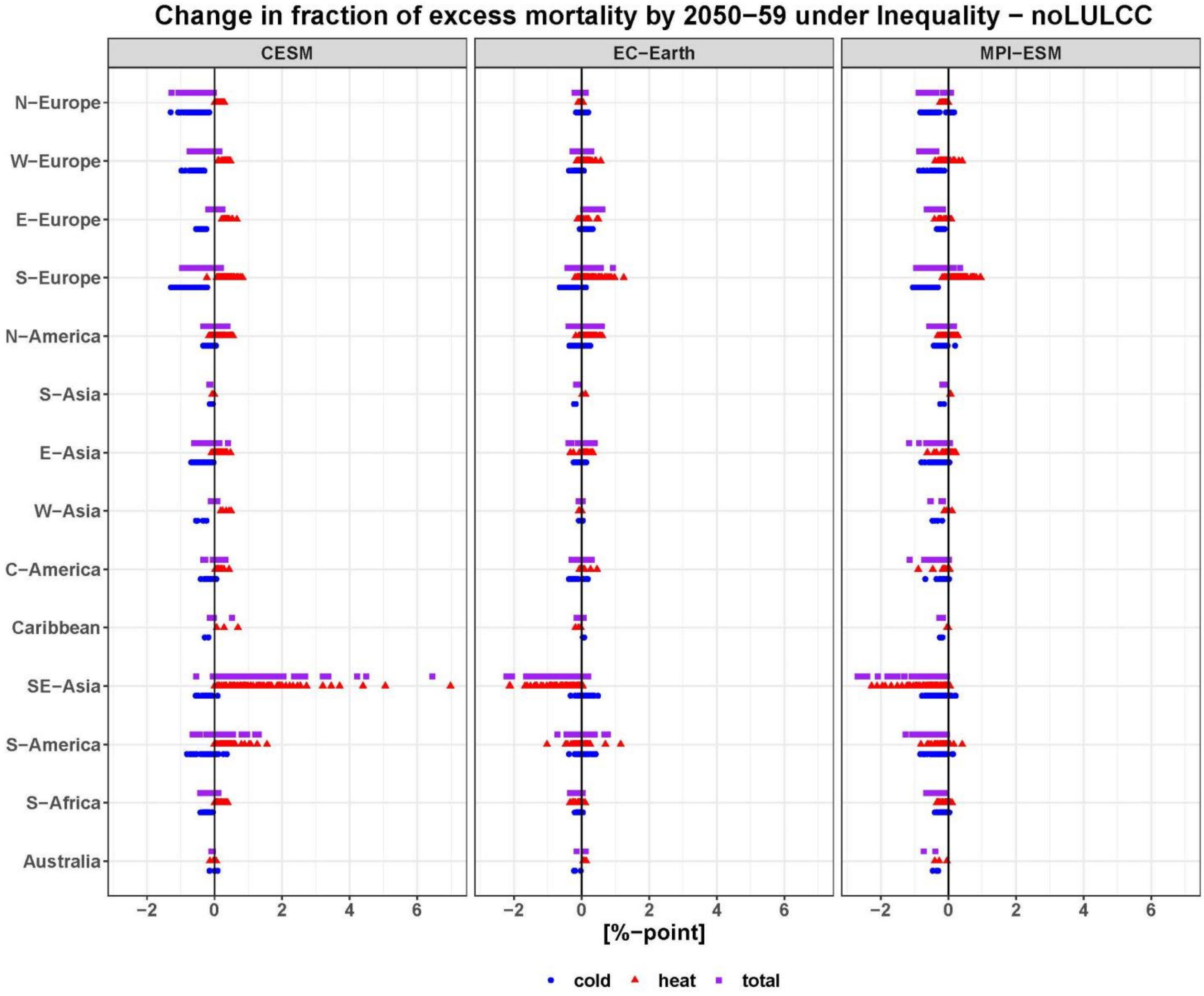

Figure S12: Change in fraction of all-cause excess cumulative mortality (in %-points) under the Inequality scenario by 2050-2059 relative to noLULCC. The blue circles, red triangles, and purple rectangles represent the location-specific cold, heat, and total excess mortality (expressed as fraction) respectively grouped by the countries or territories in the 14 geographic regions.

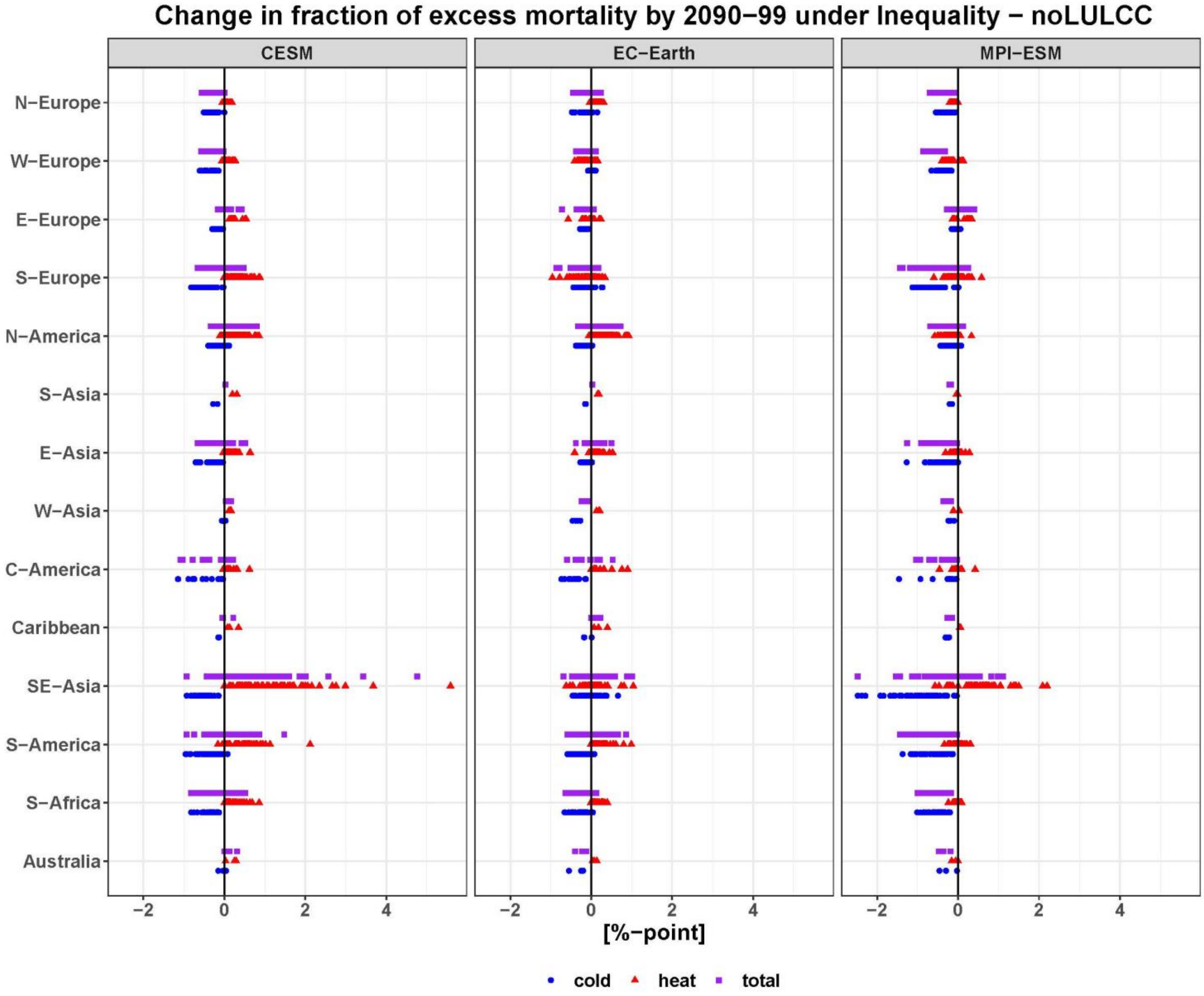

Figure S13: Same as Figure S12 but for 2090-2099.

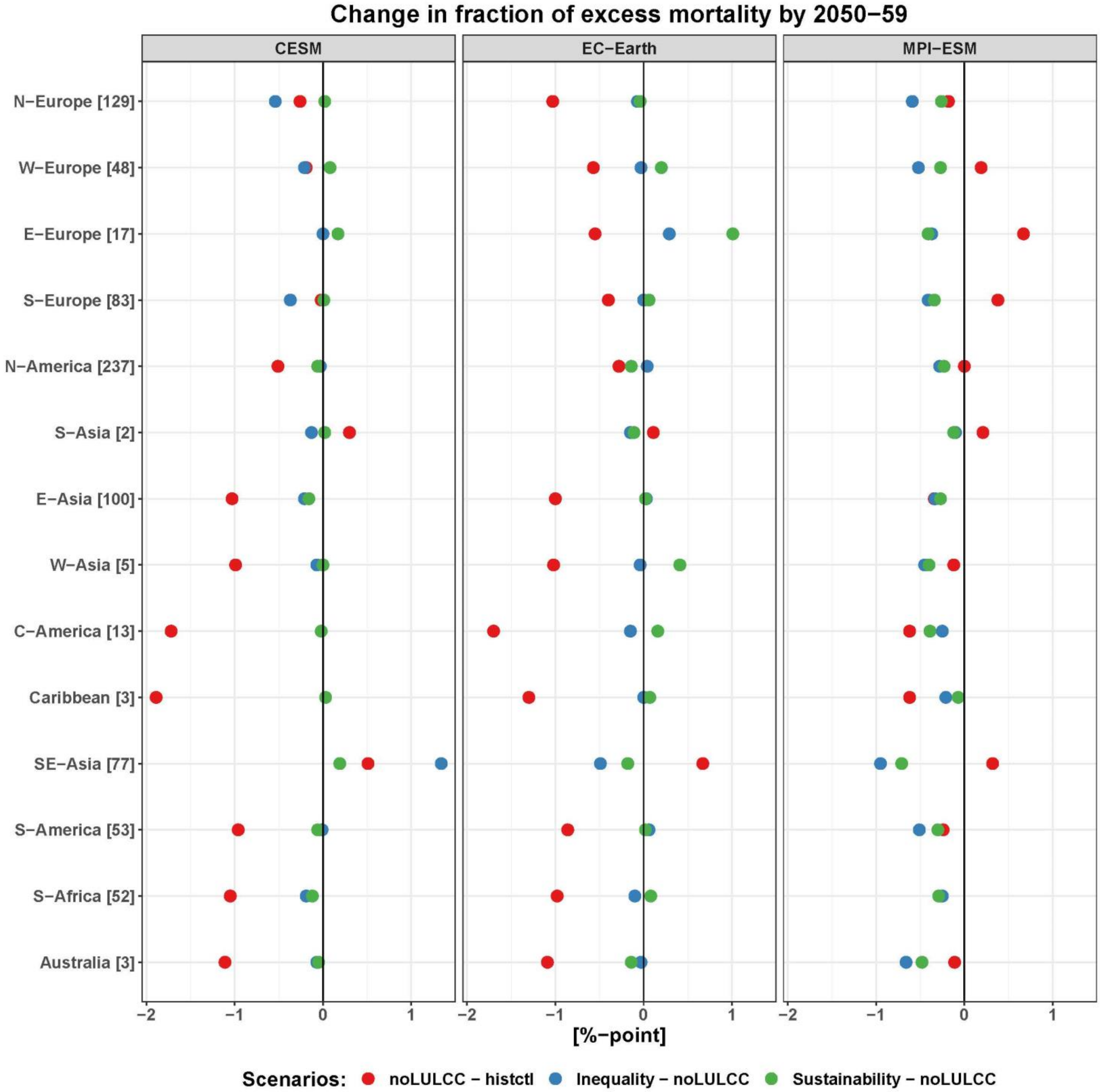

Figure S14: Regionally aggregated mean change in the fraction of total excess temperature-related mortality fractions (in %-points) by 2050-2099 for CESM, EC-Earth, and MPI-ESM. The red, blue, and green bars show the mortality impacts for the noLULCC, Inequality, and Sustainability scenarios respectively, grouped by 14 geographic regions. The numbers following the region names on the Y-axis show the number of MCC locations.

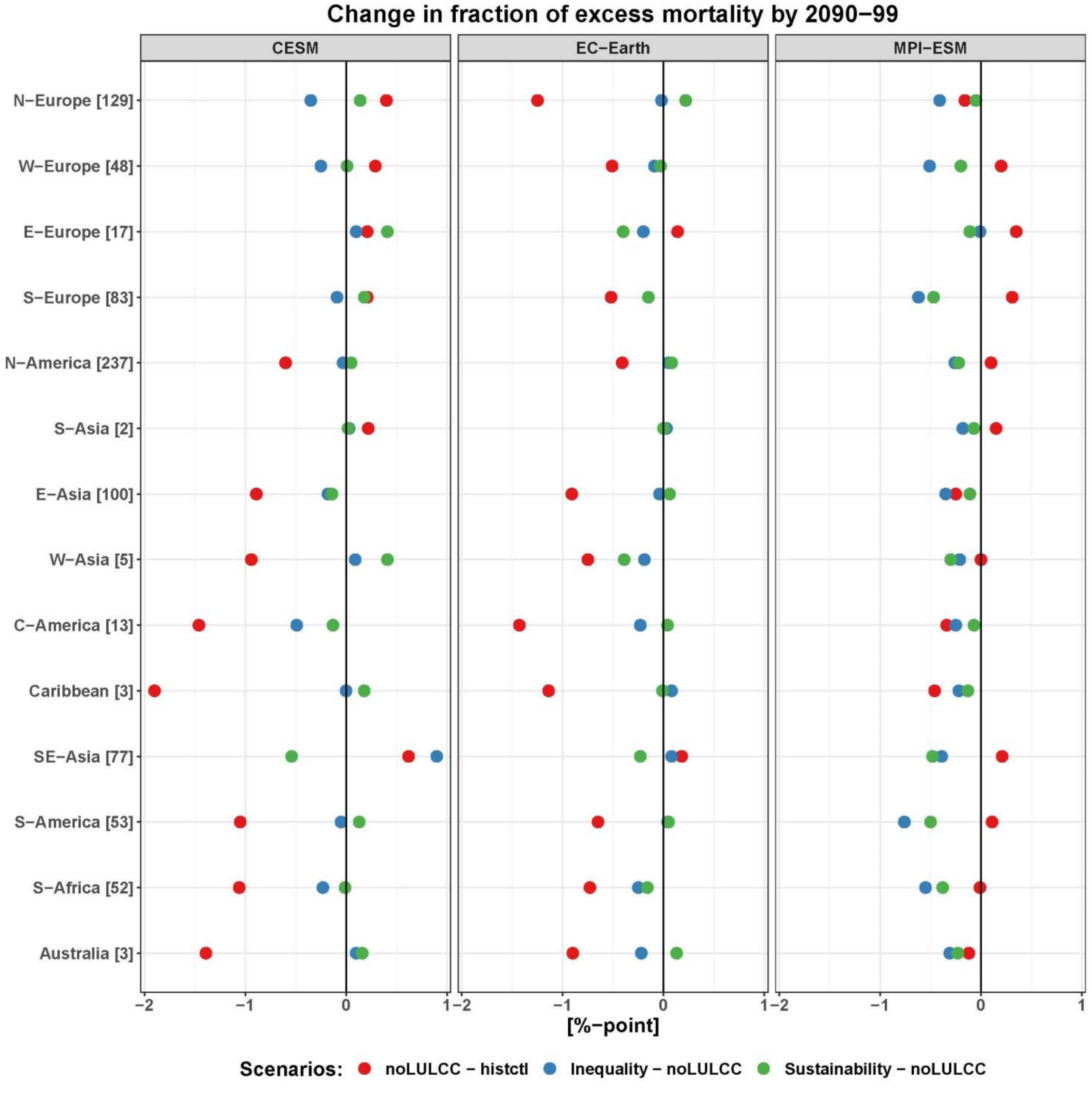

Figure S15: Same as Figure S14 but for 2090-2099.

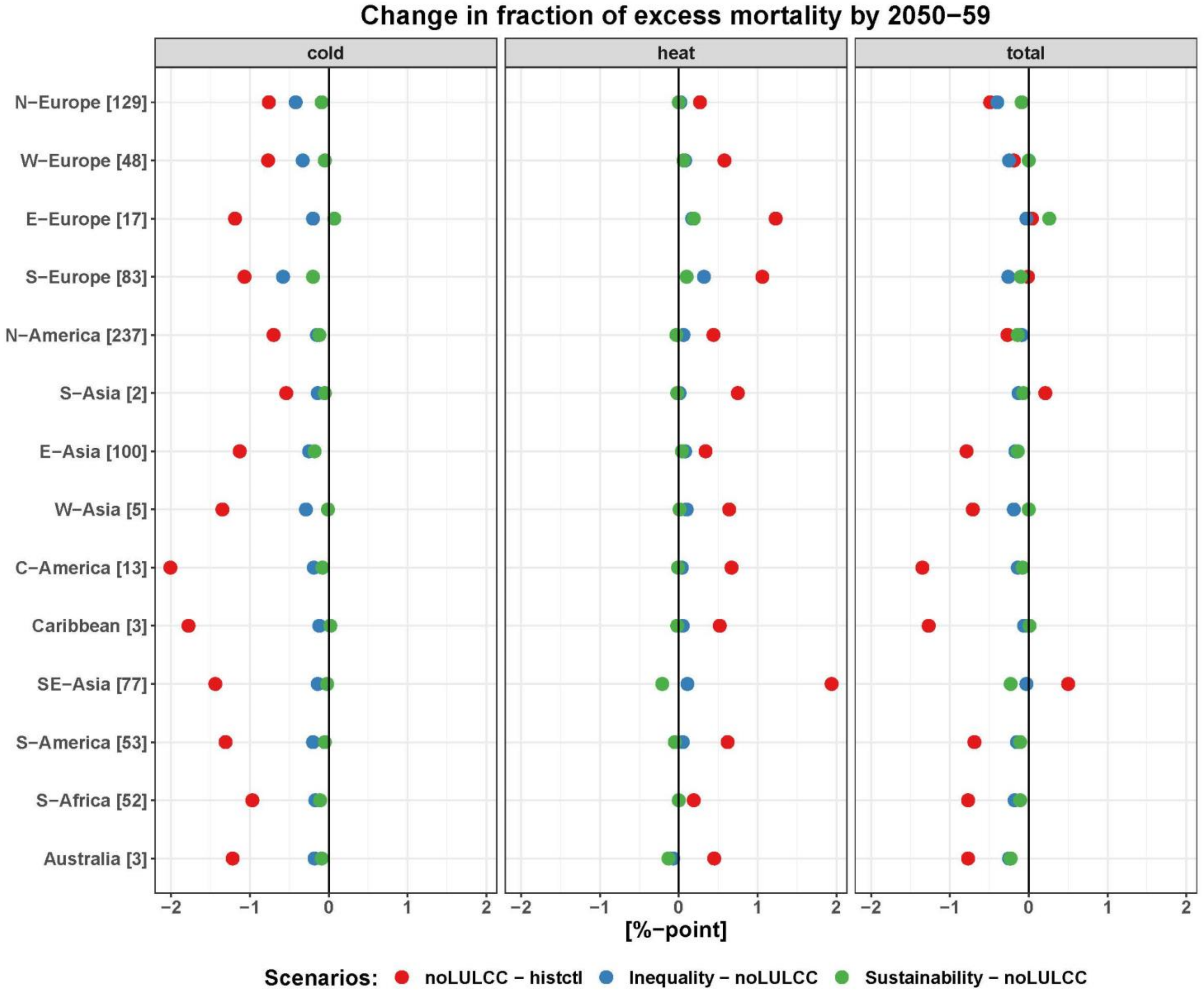

Figure S16: Regionally aggregated multi-model mean change in the fraction of excess temperature-related mortality (in %-points) by 2050-2099. The red, blue, and green bars show the mortality impacts for the noLULCC, Inequality, and Sustainability scenarios respectively, grouped by 14 geographic regions. The numbers following the region names on the Y-axis show the number of MCC locations.

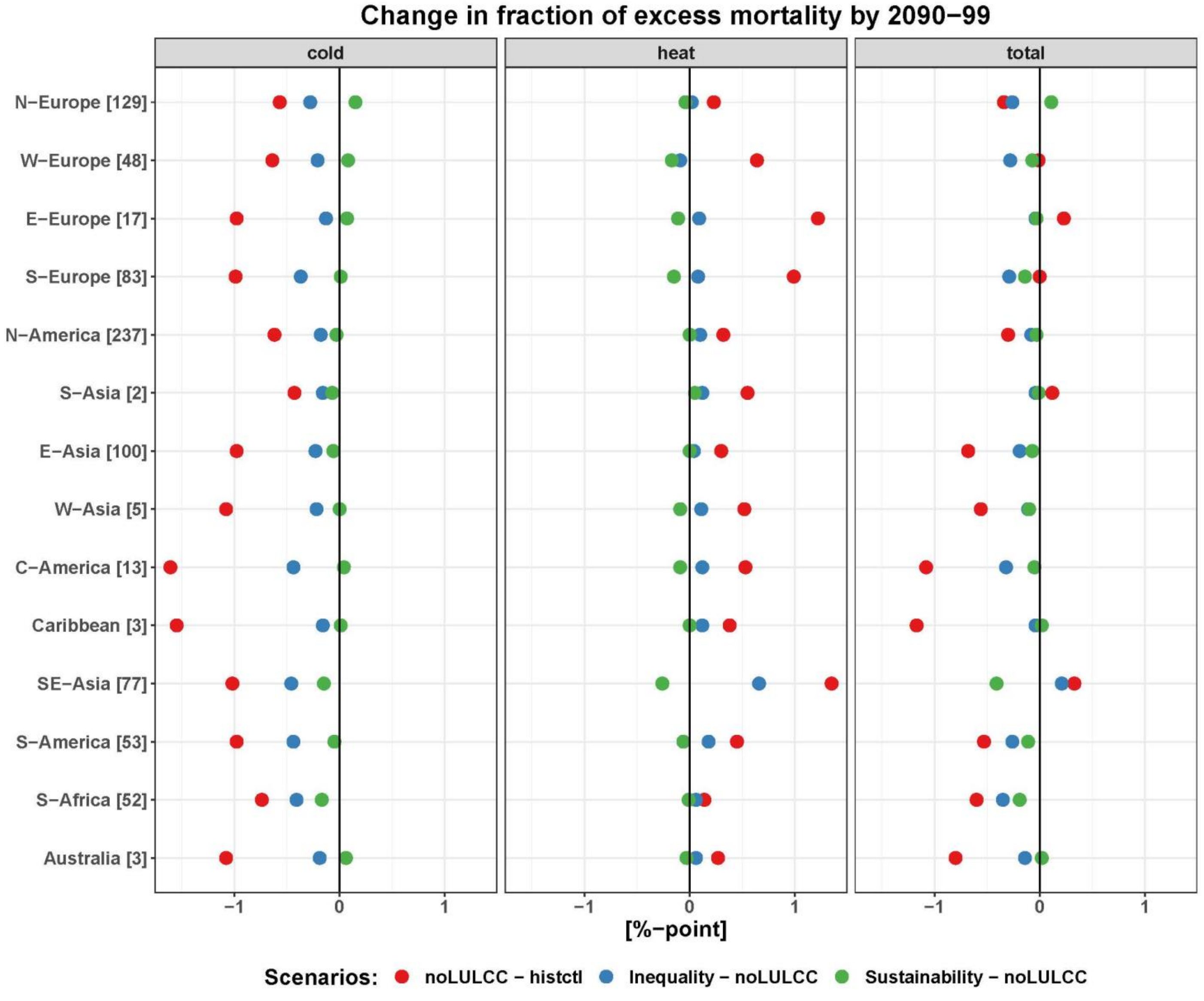

Figure S17: Same as Figure S16 but for 2090-2099.

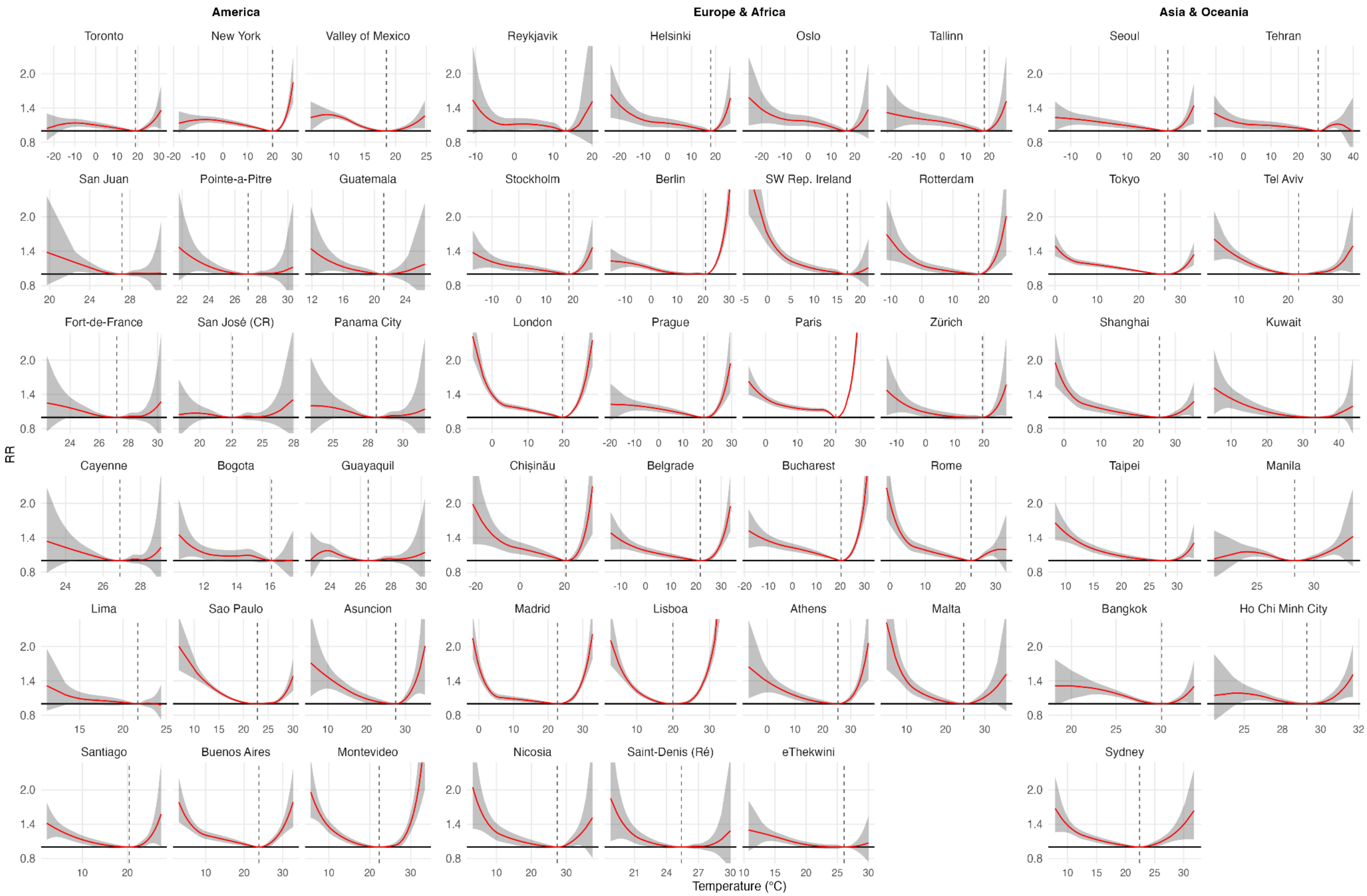

Figure S18: Overall cumulative exposure-response associations in selective cities representative of the 52 countries and territories with 95% confidence intervals

(CI) - shaded. Exposure-response associations (red) as best linear unbiased prediction (BLUP) using the distribution drawn from MCC station temperature. Dashed vertical grey lines are the minimum mortality temperatures (MMTs). RR=relative risk.

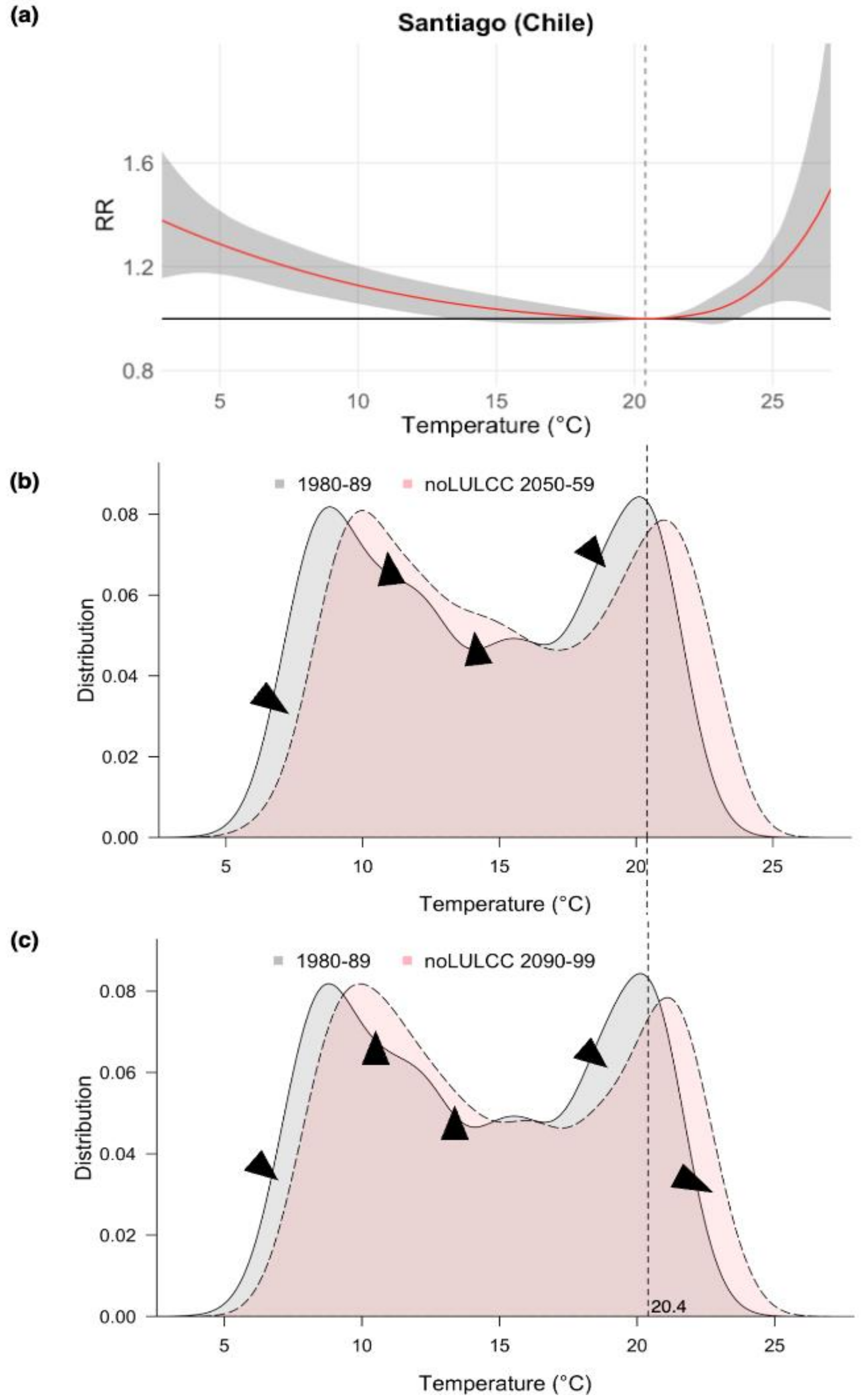

Figure S19: (a) Historical exposure-response association for Santiago (Chile). Overall cumulative exposure-response association with 95% confidence intervals (CI) -shaded (RR=relative risk, as in Fig. S18). (b) and (c) Multi-model distribution of daily mean temperature in historical (1980-89), by mid-century (2050-59) and end-century (2090-99) for Santiago under noLULCC scenario. The dashed vertical lines in all sub-plots represent the minimum mortality temperature (MMT) of Santiago (20.4 °C). The upward (downward) black triangles in (b) and (c) illustrate an increase (decrease) in the exposure of days with daily mean temperature below the MMT (i.e., cold temperatures) under noLULCC relative to historical. Comparing with the exposure-response association in (a), the change in temperature distributions would translate to a decline in future cold-related excess-deaths under noLULCC marginally outnumbering the corresponding future heat-related excess deaths, resulting in a decline in total (cold and heat) excess-deaths (Table S2).

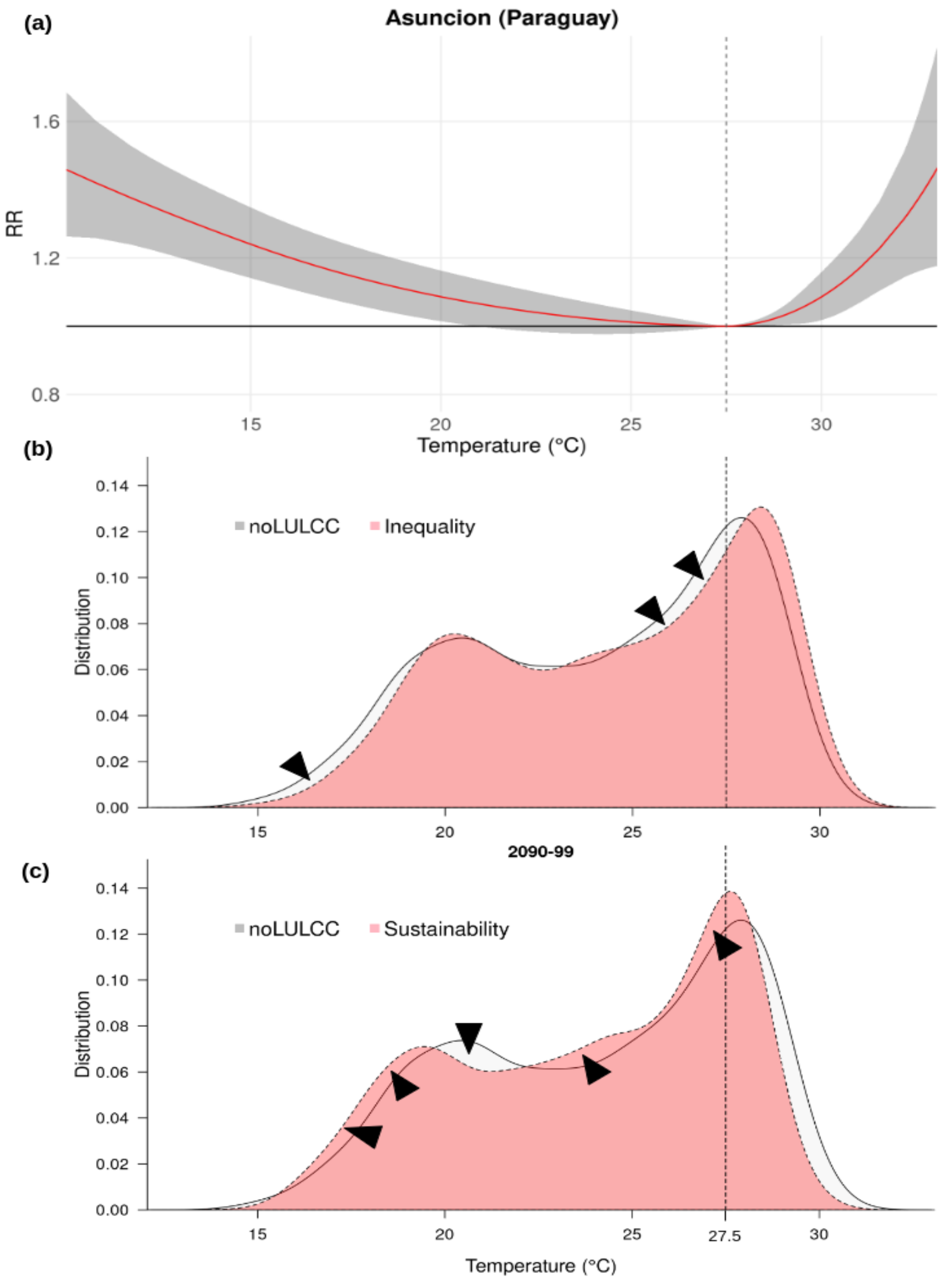

Figure S20: (a) Historical exposure-response association for Asuncion (Paraguay). Overall cumulative exposure-response association with 95% confidence intervals (CI) -shaded (RR=relative risk, as in Figs. S18). (b) and (c) Multi-model distribution of daily mean temperature by end-century (2090-99) for Asuncion under Inequality and Sustainability scenarios relative to noLULCC. The dashed vertical lines in all sub-plots represent the minimum mortality temperature (MMT) at Asuncion (27.5 °C). The upward (downward) black triangles in sub-plots (b) and (c) illustrate an increase (decline) in the exposure of days with daily mean temperature below the MMT (i.e., cold temperatures) under Sustainability (Inequality) scenario relative to noLULCC. Comparing with the exposure-response association in (a), the change in temperature distributions would translate to an increase (decline) in the cold-related excess-deaths under Sustainability (Inequality) scenario, and a marginal increase (decline) in the heat-related deaths in Inequality (Sustainability) relative to noLULCC scenario, with a total change in excess-deaths as a net effect of cold and heat-related outcomes.

Table S1: Description of the observed temperature (°C) and mortality data for the MCC locations used in the study. Where not indicated otherwise, the mean daily temperature is computed as the 24-hour average based on hourly measurements from weather station.

Country	No. and Type of Locations	Time Period (Years)	Mortality Data (Type – Source)	Meteorological Data (Source and Remarks)
Argentina	3 cities	2005-2015 (11)	Non-external causes only (ICD-9: 0-799; ICD-10: A00-R99) - National Ministry of Health (Ministerio de Salud de la Nación)	Weather station located within each city - Servicio Meteorológico Nacional (National Weather Service)
Australia	3 cities	1988-2009 (22)	Non-external causes only (ICD-9: 0-799; ICD-10: A00-R99) - Australian Bureau of Statistics	Weather stations located within ≤30 km of each city – Australian Bureau of Meteorology
Brazil	18 cities	1997-2018 (22)	Non-external causes only (ICD-9: 0-799; ICD-10: A00-R99) - Ministry of Health	Weather stations located within the urban area - National Institute of Meteorology of Brazil
Canada	26 (25 census metropolitan areas -CMA- and 1 city)	1986-2015 (30)	All causes - Canadian Mortality Database	Nearest weather station - Environment Canada
Chile	4 cities	2004-2014 (11)	All causes - Department of Statistics and Information, Ministry of Health (Departamento de Estadísticas e Información de Salud, Ministerio de Salud)	Nearest weather station - Ministerio del Medio Ambiente, Sistema de Información Nacional de Calidad del Aire (SINCA)
China	14 cities	1996-2015 (30)	Non-external causes only (ICD-9: 0-799; ICD-10: A00-R99) - Municipal Center for Disease Control and Prevention in each city	Weather station located within each city - China Meteorological Data Sharing Service System (http://data.cma.cn/)
Colombia	5 cities	1998-2013 (16)	All causes - National Administrative Department of Statistics (DANE)	Nearest weather station - Instituto de Hidrología, Meteorología y Estudios Ambientales de Colombia (IDEAM)
Costa Rica	1 city	2000-2017 (18)	All causes - Instituto Nacional de Estadística y Censo. Open Access.	Meteorological data - World Meteorological Orgnaization – National Oceanic and Atmospheric Administration (WMO-NOAA) Surface Data Hourly Global (DS3505)
Cyprus	5 cities	2004-2019 (16)	Non-external causes (ICD10: A00-R99) - Health Monitoring Unit, Ministry of Health.	Weather station located within each city – Cyprus Department of Meteorology, Ministry of Agriculture, Rural Development and Environment
Czech Republic	4 cities	1994-2020 (27)	All causes - Czech Statistical Office and the Institute of Health Information and Statistics	Weather station located within each city - Czech Hydrometeorological Institute (measurements in standard climatic terms 7:00, 14:00 and 21:00 local time, and daily means)
Ecuador	2 cities	2014-2018 (5)	All causes - *Estonian Causes of Death Registry*	Meteorological data - WMO-NOAA Surface Data Hourly Global (DS3505)

Estonia	5 cities	1997-2020 (24)	All causes - Estonian Causes of Death Registry	Nearest weather station - Estonian Environment Agency
Finland	1 city	1994-2014 (21)	All causes - Statistics Finland	Mean daily temperature - Finnish Meteorological Institute. The weather stations around the country were interpolated onto a 10×10 km grid covering the whole of Finland, using a Kriging model
France (FR)	20 cities	2000-2017 (18)	All causes - French National Institute of Health and Medical Research (CepiDC)	Nearest weather station, usually the airport - Meteo France
French Guiana (FR)	1 city	2000-2015 (16)	Same as France	Same as France
Germany	15 cities	1993-2020 (28)	All causes - Research Data Centres of the Federation and the Federal States of Germany (Forschungsdatenzentrum der Statistischen Ämter des Bundes und der Länder)	Nearest weather station - Climate Data Centre of the German National Meteorological Service (Deutscher Wetterdienst)
Greece	1 city	2001-2010 (10)	All causes - Hellenic Statistical Authority	National observatory of Athens (http://www.noa.gr/) from site "Thisio" located in the city of Athens.
Guadeloupe (FR)	1 city	2000-2015 (16)	Same as France	Same as France
Guatemala	1 city	2009-2016 (8)	All causes - Instituto Nacional de Estadística, Unidad de Estadística de Salud.	Nearest weather station - Instituto Nacional de Sismología, Vulancología, Meteorología y Hidrología.
Iceland	1 city	2000-2018 (19)	All causes - Statistics Iceland	Weather station data - Reykjavik (capital area) provided by the Icelandic Meteorological Office, Reykjavik (www.vedur.is)
Iran	2 cities	2002-2015 (14)	All causes - Ferdows organization of Mashhad Municipality	Nearest weather station - IRAN Meteorological Organization (IRIMO) (http://www.irimo.ir)
Island of Ireland	Total 6 regions: 4 covering all Island population (ROI) and 2 in the Northern Ireland (NI)	1985-2007 (23)	Non-external causes only (ICD-9: 0- 799; ICD-10: A00-R99) provided by Irish Central Statistics Office Northern and Ireland Social Research Agency.	Two weather stations for each ROI regions and NI regions from Met Eireann, and the United Kingdom Meteorological Office (UKMO)
Israel	4 cities	1985-2020 (36)	All causes – Israel Central Bureau of Statistics	Israel Meteorological Service (most representative station for each region/city, selected after consulting with the meteorological service experts).
Italy	18 cities	2006-2015 (10)	All causes - local mortality registries and the rapid mortality surveillance system	Airport monitoring station located closest to the city centre - Meteorological Service of the Italian Air Force. 24-h average based on 6-h measurements

Japan	47 prefectures	1985-2020 (36)	All causes - Ministry of Health, Labour and Welfare	Weather station located within the urban area of the capital city - Japan Meteorology Agency (JMA)
Kuwait	1 city	2000-2016 (17)	Non-external causes only (ICD-9: 0-799; ICD-10: A00-R99) - National Center for Health Information, Ministry of Health	Nearest weather station – the Directorate General of Civil Aviation (Kuwait Airport) and Kuwait's Environmental Public Authority.
Malta	1 city	1995-2019 (25)	All causes - Directorate of Health Information and Research in Malta.	Weather data collected from Malta’s only weather station situated at Luqa were obtained from the global surface archives of WeatherGraphics.com in the form of 3-hourly machine-coded SYNOP data (WMO FM 14)
Martinique (FR)	1 city	2000-2015 (16)	Same as France	Same as France
Mexico	10 cities	1998-2014 (17)	All causes - National Institute of Statistics, Geography and Informatics	Weather station located within the urban area or at a near airport - Primarily Servicio Meteorológico Nacional (SMN) Estaciones Sinópticas Meteorológicas (ESIMES), Estaciones Meteorológicas Automáticas (EMAS)* and Observatories. Otherwise (i) Instituto Nacional de Ecología y Cambio Climático (INECC). (ii) Red de Meteorología y Radiación Solar (REDMET) from the Sistema de Monitoreo Atmosférico de la Ciudad de México (SIMAT). (iii) WMO stations from the Weather Underground.)
Moldova	4 cities	2001-2010 (10)	All causes - National Centre for Health Management	Nearest weather station - State Hydrometeorological Service, Moldova. Mean daily temperature computed as the average between daily minimum and maximum
The Netherlands	5 cities	1995-2016 (22)	All causes - Statistics Netherlands	Nearest weather station – Royal Dutch Meteorological Institute (KNMI)
Norway	1 city	1985-2018 (34)	All causes - Norwegian Cause of Death registry	Mean daily temperature based on the observational modelled dataset from the Norwegian Meteorological Institute.
Panama	1 city	2013-2016 (4)	All causes Instituto Nacional de Estadística y Censo, Centro de Información Estadística.	Open access temperature data - Empresa de Transmisión Eléctrica, S.A. (ETESA)
Paraguay	1 city	2004-2019 (16)	All causes - Ministerio de Salud Pública y Bienestar Social, Dirección General de Información Estratégica en Salud, Subsistema de Información de Estadísticas Vitales	Meteorological data - WMO-NOAA Surface Data Hourly Global (DS3505)
Peru	18 regions	2008-2014 (7)	All causes provided by the Peruvian Ministry of Health (MINSA in Spanish)	Mean daily temperature (in ˚C) was obtained from the National Meteorology and Hydrology

				Service of Peru (SENAMHI in Spanish). A total of 18 weather stations (one station per region) contributed data to each department series.
The Philippines	13 cities	2006-2019 (14)	All causes - Philippine Statistics Agency	Station in or near location - Philippine Atmospheric Geophysical and Astronomical Services Administration
Portugal	6 cities	1985-2018 (34)	All causes - Statistics Portugal	Meteorological data - WMO-NOAA Surface Data Hourly Global (DS3505)
Puerto Rico	1 city	2009-2016 (8)	All causes - Instituto de Estadísticas Vitales de Puerto Rico, Área de Estadísticas Vitales del Departamento de Salud	Meteorological data - WMO-NOAA Surface Data Hourly Global (DS3505)
La Réunion (FR)	1 city	2000-2015 (16)	Same as France	Same as France
Romania	8 cities	1994-2016 (23)	All causes - Romanian National Institute of Statistics	Mean daily temperature - National Meteorological Administration of Romania (NMARO, accessed from https://www.ecad.eu/)
Serbia	1 city	1995-2021 (27)	All causes - Statistical Office of the Republic of Serbia	Meteorological data - WMO-NOAA Surface Data Hourly Global (DS3505)
South Africa	52 districts municipalities	1997-2013 (17)	All causes – Statistics South Africa	Meteorology – weather station data from the National Oceanographic and Atmospheric Association and the South African Agricultural Research Council
South Korea	36 cities	1997-2018 (22)	All causes – South Korea Bureau of Statistics	Weather station located within the location - Korea Meteorological Administration
Spain	52 cities	1990-2014 (25)	Non–external causes (ICD–9: 0–799; ICD–10: A00–R99) - Spain National Institute of Statistics.	Weather station in the location or at a near airport - Spain National Meteorology Agency
Sweden	3 cities	1990-2016 (27)	All causes - Swedish Cause of Death Register at the Swedish National Board of Health and Welfare	Weather station in the location or at a near airport (Environment and Health Administration)
Switzerland	8 (7 cities and 1 metropolitan area -Lugano)	1995-2013 (19)	Non–external causes only other than accidents (ICD–10codes A00–R99, V01–V99, W00–X59) - Federal Office of Statistics	Weather station located within or near the urban area for each city - IDAWEB database (a service provided by MeteoSwiss, the Swiss Federal Office of Meteorology and Climatology).
Taiwan	3 cities	1994-2014 (21)	All causes - Department of Health	1-15 stations per location - Taiwan Environmental Protection Administration
Thailand	62 regions	1999-2008 (10)	Non-external (ICD-9: 0- 799; ICD-10: A00-R99) mortality, provided the Ministry of Public Health, Thailand.	Mean daily temperature (in ˚C) computed as the average between daily minimum and maximum, were obtained from the Meteorological Department, Ministry of Information and Communication Technology, Thailand.
UK	112 cities	1990-2020 (31)	All causes - Office of National Statistics.	29 stations on average per location - British Atmospheric Data Centre (BADC)

Uruguay	1 city	2012-2016 (5)	Non-external causes - Ministerio de Salud Publica (MSP)	Nearest weather station - Instituto Uruguayo de Meteorología (INUMET)
USA	211 cities	1985-2006 (22)	All causes - National Center for Health Statistics	Weather station closest to the city centre - National Climatic Data Center (NCDC) of NOAA
Vietnam	2 cities	2009-2013 (5)	All causes - Provincial Department of Health	Weather station at city airport

Table S2: Detailed summary statistics.

See **Supplementary Table S2.xlsx**

Table S3: Changes in global mean temperature by ESM and scenario.

**ESM**	**CESM**	**EC-Earth**	**MPI-ESM**
noLULCC by 2069-2099 relative to 1985-2014	+1.05°C	+1.02°C	+0.35°C
Inequality-noLULCC by 2069-2099	+0.29°C	+0.29°C	+0.17°C
Sustainability-noLULCC by 2069-2099	-0.07°C	+0.07°C	+0.05°C

MCC Collaborative Research Network†.

Rosana Abrutzky, Yuming Guo, Shilu Tong, Micheline de Sousa Zanotti Stagliorio Coelho, Paulo Hilario Nascimento Saldiva, Patricia Matus Correa, Nicolás Valdés Orteg, Haidong Kan, Samuel Osorio, Jan Kyselý, Aleš Urban, Hans Orru, Ene Indermitte, Jouni J. K. Jaakkola, Niilo Ryti, Mathilde Pascal, Alexandra Schneider, Veronika Huber, Klea Katsouyanni, Antonis Analitis, Hanne Krage Carlsen, Fatemeh Mayvaneh, Hematollah Roradeh, Patrick Goodman, Ariana Zeka, Raanan Raz, Paola Michelozzi, Francesca de'Donato, Masahiro Hashizume, Yoonhee Kim, Barrak Alahmad, Magali Hurtado Diaz, Eunice Elizabeth Félix Arellano, Ala Overcenco, Danny Houthuijs, Caroline Ameling, Shilpa Rao, Gabriel Carrasco, Xerxes Seposo, Paul Lester Carlos Chua, Susana das Neves Pereira da Silva, Joana Madureira, Iulian-Horia Holobaca, Noah Scovronick, Fiorella Acquaotta, Ho Kim, Whanhee Lee, Aurelio Tobias, Carmen Íñiguez, Bertil Forsberg, Martina S. Ragettli, Yue Leon Guo, Shih-Chun Pan, Shanshan Li, Valentina Colistro, Antonella Zanobetti, Joel Schwartz, Tran Ngoc Dang, Do Van Dung and John Paul Cauchi.

References

Gasparrini, A., 2014. Modeling exposure-lag-response associations with distributed lag non-linear models. Stat. Med. 33, 881–899. https://doi.org/10.1002/sim.5963

Gasparrini, A., Armstrong, B. & Kenward, M. G., 2012. Multivariate meta- analysis for non- linear and other multi- parameter associations. *Statistics in Medicine* **31**, 3821–3839. https://doi.org/10.1002/sim.5471

Gasparrini, A., Armstrong, B., 2013. Reducing and meta-analysing estimates from distributed lag non-linear models. BMC Med. Res. Methodol. 13, 1. https://doi.org/10.1186/1471-2288-13-1

Gasparrini, A., Guo, Y., Hashizume, M., Lavigne, E., Zanobetti, A., Schwartz, J., Tobias, A., Tong, S., Rocklöv, J., Forsberg, B., Leone, M., Sario, M.D., Bell, M.L., Guo, Y.-L.L., Wu, C., Kan, H., Yi, S.-M., Coelho, M. de S.Z.S., Saldiva, P.H.N., Honda, Y., Kim, H., Armstrong, B., 2015. Mortality risk attributable to high and low ambient temperature: a multicountry observational study. The Lancet 386, 369–375. https://doi.org/10.1016/S0140-6736(14)62114-0

Gasparrini, A., Guo, Y., Sera, F., Vicedo-Cabrera, A.M., Huber, V., Tong, S., de Sousa Zanotti Stagliorio Coelho, M., Nascimento Saldiva, P.H., Lavigne, E., Matus Correa, P., Valdes Ortega, N., Kan, H., Osorio, S., Kyselý, J., Urban, A., Jaakkola, J.J.K., Ryti, N.R.I., Pascal, M., Goodman, P.G., Zeka, A., Michelozzi, P., Scortichini, M., Hashizume, M., Honda, Y., Hurtado-Diaz, M., Cesar Cruz, J., Seposo, X., Kim, H., Tobias, A., Iñiguez, C., Forsberg, B., Åström, D.O., Ragettli, M.S., Guo, Y.L., Wu, C., Zanobetti, A., Schwartz, J., Bell, M.L., Dang, T.N., Van, D.D., Heaviside, C., Vardoulakis, S., Hajat, S., Haines, A., Armstrong, B., 2017. Projections of temperature-related excess mortality under climate change scenarios. Lancet Planet. Health 1, e360–e367. https://doi.org/10.1016/S2542-5196(17)30156-0

Hempel S, Frieler K, Warszawski L, Schewe J, Piontek F. A trend-preserving bias correction - the ISI-MIP approach. Earth Syst Dyn. 2013;4(2):219-236. https://doi.org/10.5194/esd-4-219-2013

Honda Y, Kondo M, McGregor G, Kim H, Guo YL, Hijioka Y, Yoshikawa M, Oka K, Takano S, Hales S, Kovats RS =, 2014: Heat-related mortality risk model for climate change impact projection. Environ Health Prev Med, 19, 56–63, https://doi.org/10.1007/s12199-013-0354-6

Sera, F., Armstrong, B., Blangiardo, M., Gasparrini, A., 2019. An extended mixed-effects framework for meta-analysis. Stat. Med. 38, 5429–5444. https://doi.org/10.1002/sim.8362
